# Supplementary material for: Comprehensive Genome-Wide Analysis and Expression Pattern Profiling of the SlHVA22 Gene Family Unravels Their Likely Involvement in the Abiotic Stress Adaptation of Tomato
Source: Int J Mol Sci. 2022 Oct 13;23(20):12222. doi: 10.3390/ijms232012222 (PMC9602767; doi:10.3390/ijms232012222)
Supplement: Supplementary file 1 [file ijms-23-12222-s001.zip › Supplementary materials.pdf]

Supplementary Materials: Comprehensive genome-wide analysis and expression pattern profiling of the *SlHVA22* gene family unravels their likely involvement in the abiotic stress adaptation of tomato

Antt Htet Wai, Muhammad Waseem, Lae-Hyeon Cho, Sang-Tae Kim, Do-jin Lee, Chang-Kil Kim and Mi-Young Chung*

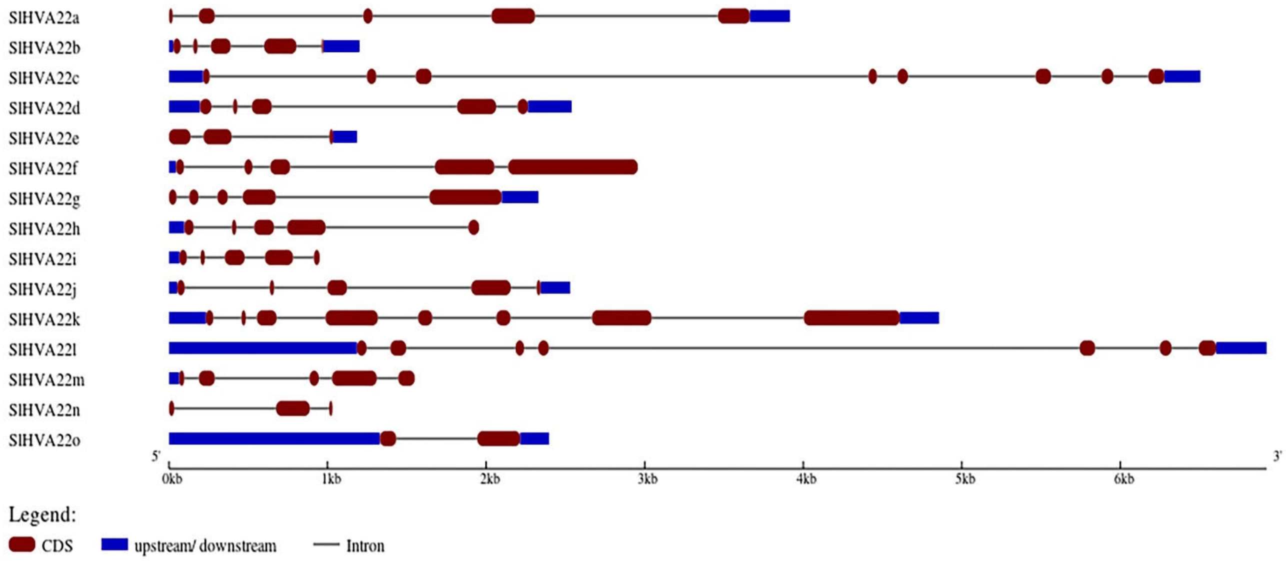

Figure S1. Schematic depiction of the exon-intron structure of *SlHVA22* genes. Dark red boxes represent exons, black lines indicate introns, and blue boxes denote untranslated regions.

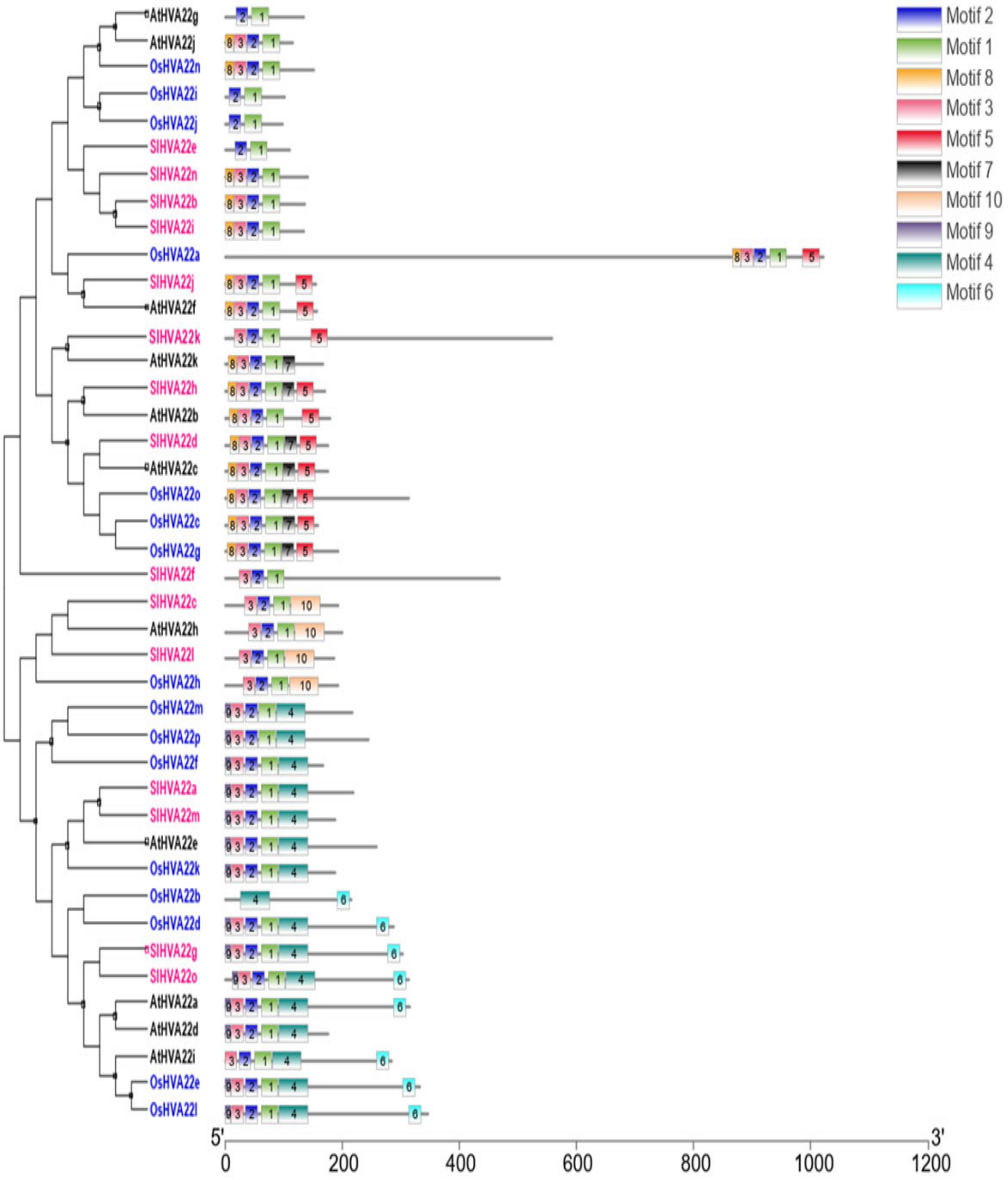

Figure S2. Conserved motif analysis of HVA22 proteins from tomato, *Arabidopsis* and rice. Motif 1 and motif 2 are located in the TB2/DP1/HVA22 domain. Different colored boxes signify different motifs, with the motif names in the center.

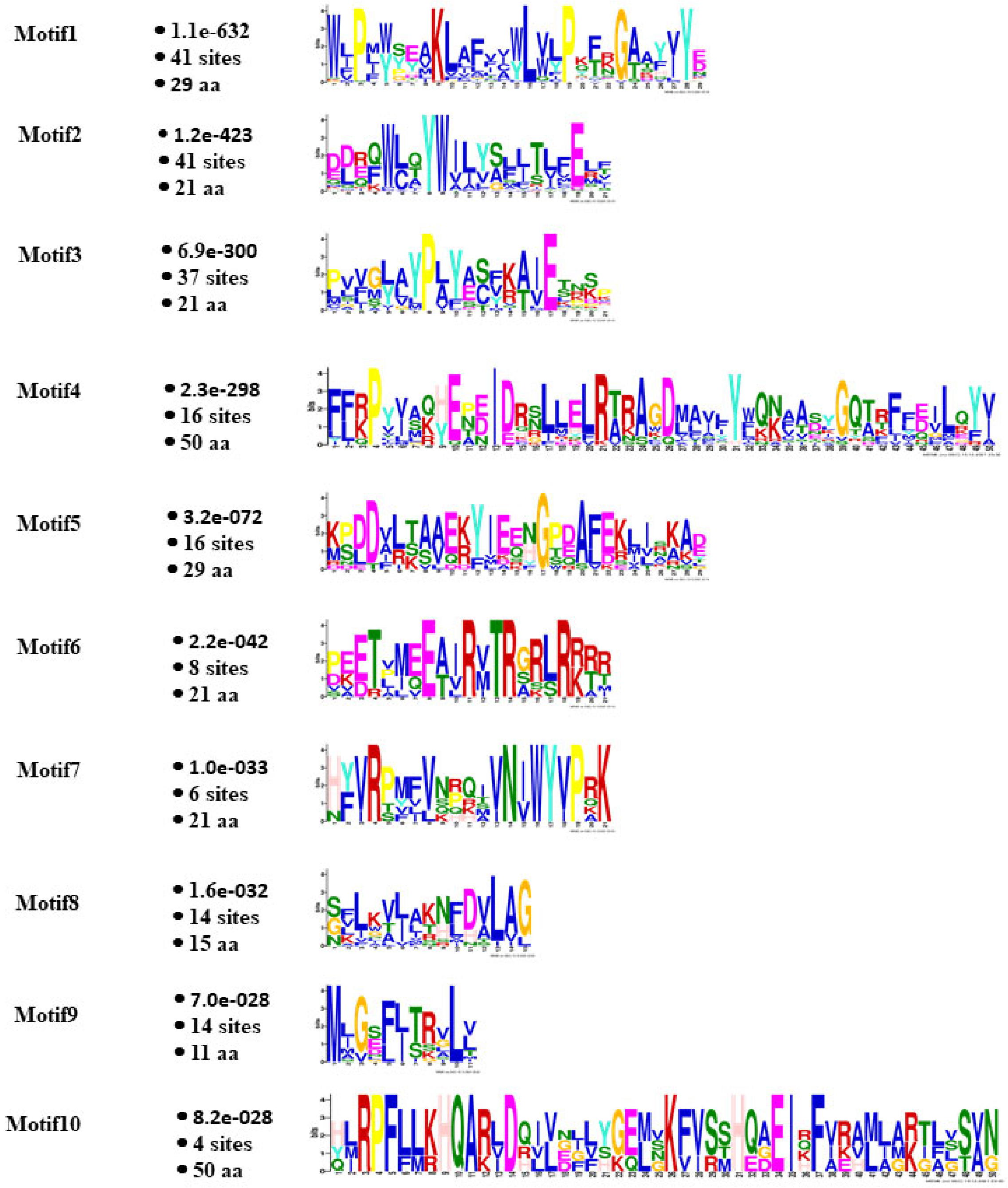

Figure S3. Overview of conserved motifs of HVA22 proteins from tomato, *Arabidopsis* and rice.

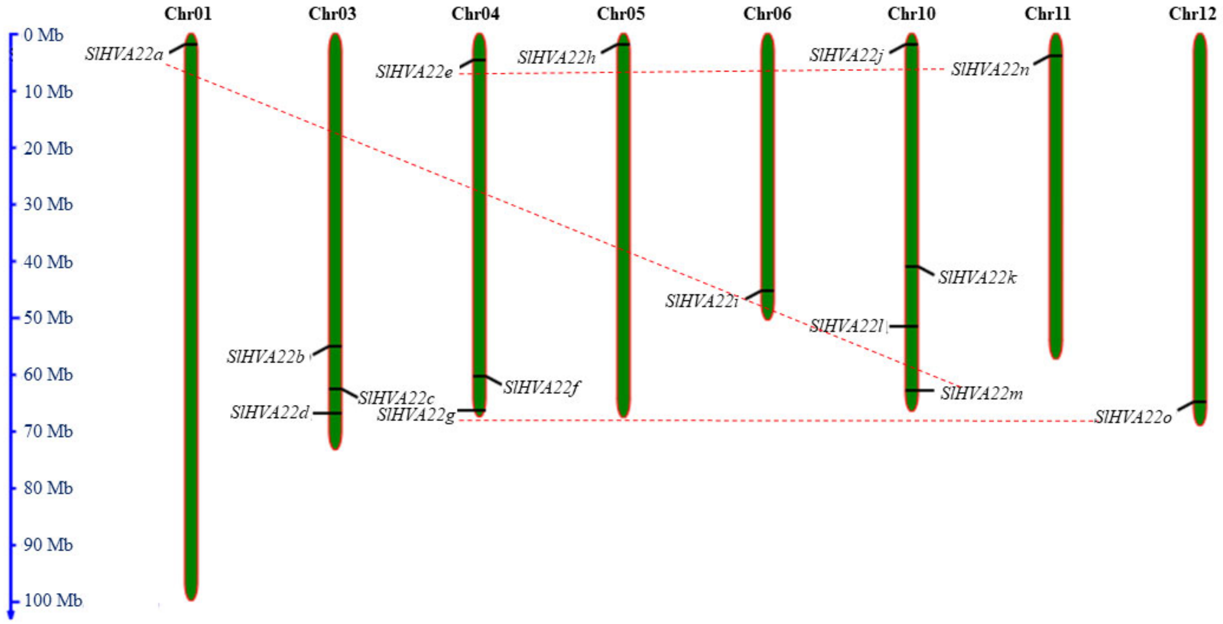

Figure S4. Chromosome locations of *SlHVA22* genes. The chromosome numbers are labelled at the top of chromosomes and the length of chromosomes can be estimated by the scale bar on the left shown in megabases (Mb). The duplicated gene pairs are linked by the red dotted lines.

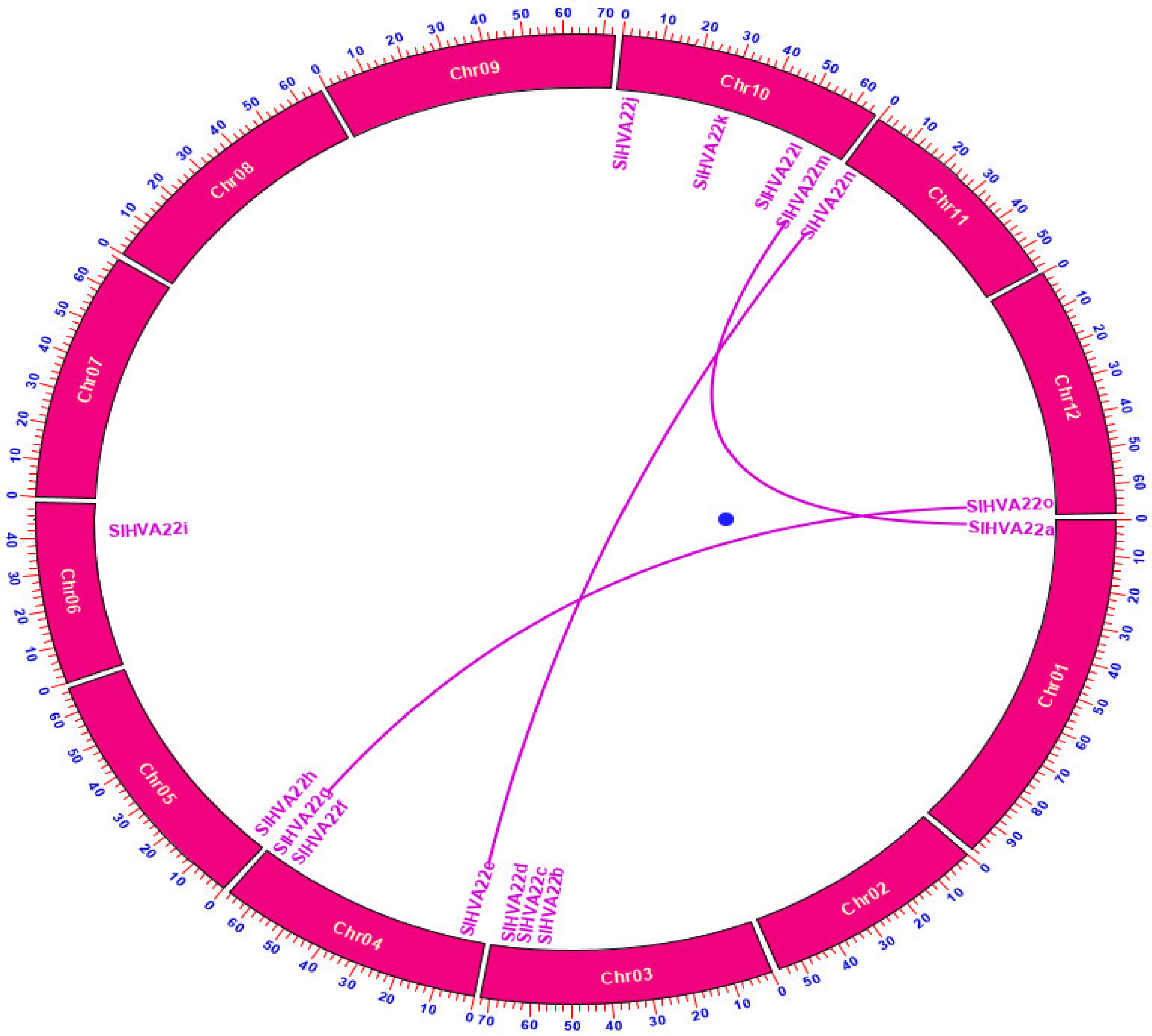

Figure S5. Gene duplication analysis of *HVA22* genes in tomato. Chromosome numbers and gene positions are indicated and the length of each chromosome is displayed on a megabase scale (Mb). The segmentally duplicated gene pairs are interconnected by pink red lines.

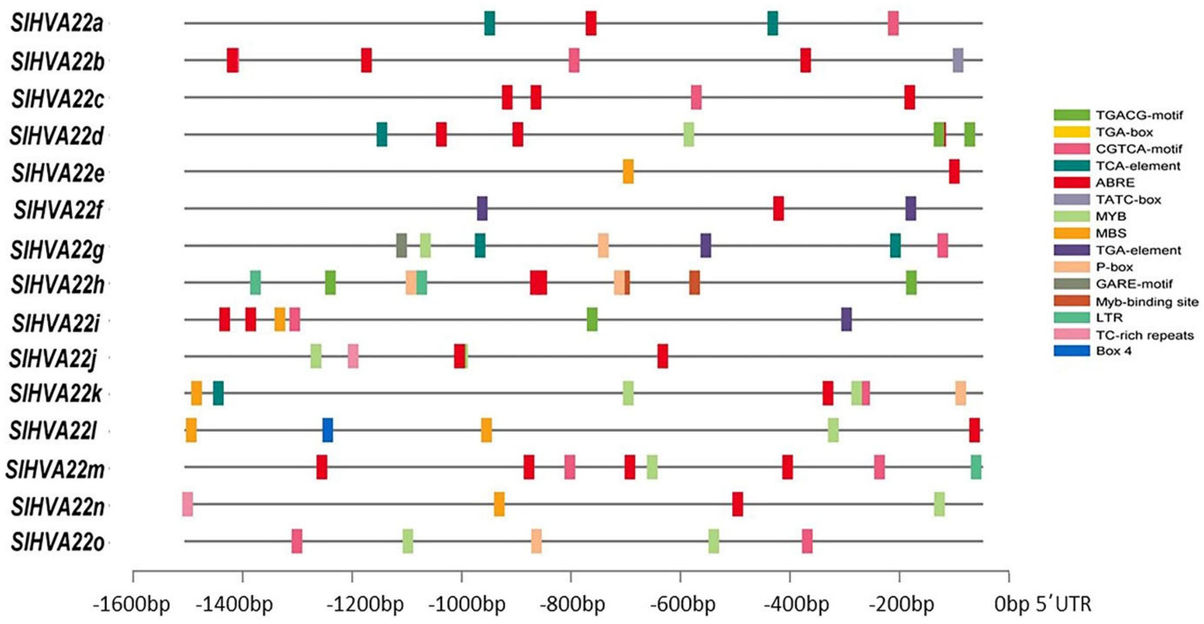

Figure S6. Predicted *cis*-acting elements in the promoters of *SlHVA22* genes. The identified elements are: drought-responsive MYB-binding site (MBS) and MYB elements, defense and stress-responsive elements (TC-rich repeats), jasmonic acid-responsive elements (CGTCA-motif), TCA-elements (related to the SA response), auxin responsive elements (TGA-element and TGA-box), ABA-responsive elements (ABRE), low-temperature-responsive elements (LTR), and P-box, GARE- and TATC-elements associated with GA response. The scale denotes the positions of cis-regulatory elements in regard to the translation start site (taken as +1 bp).

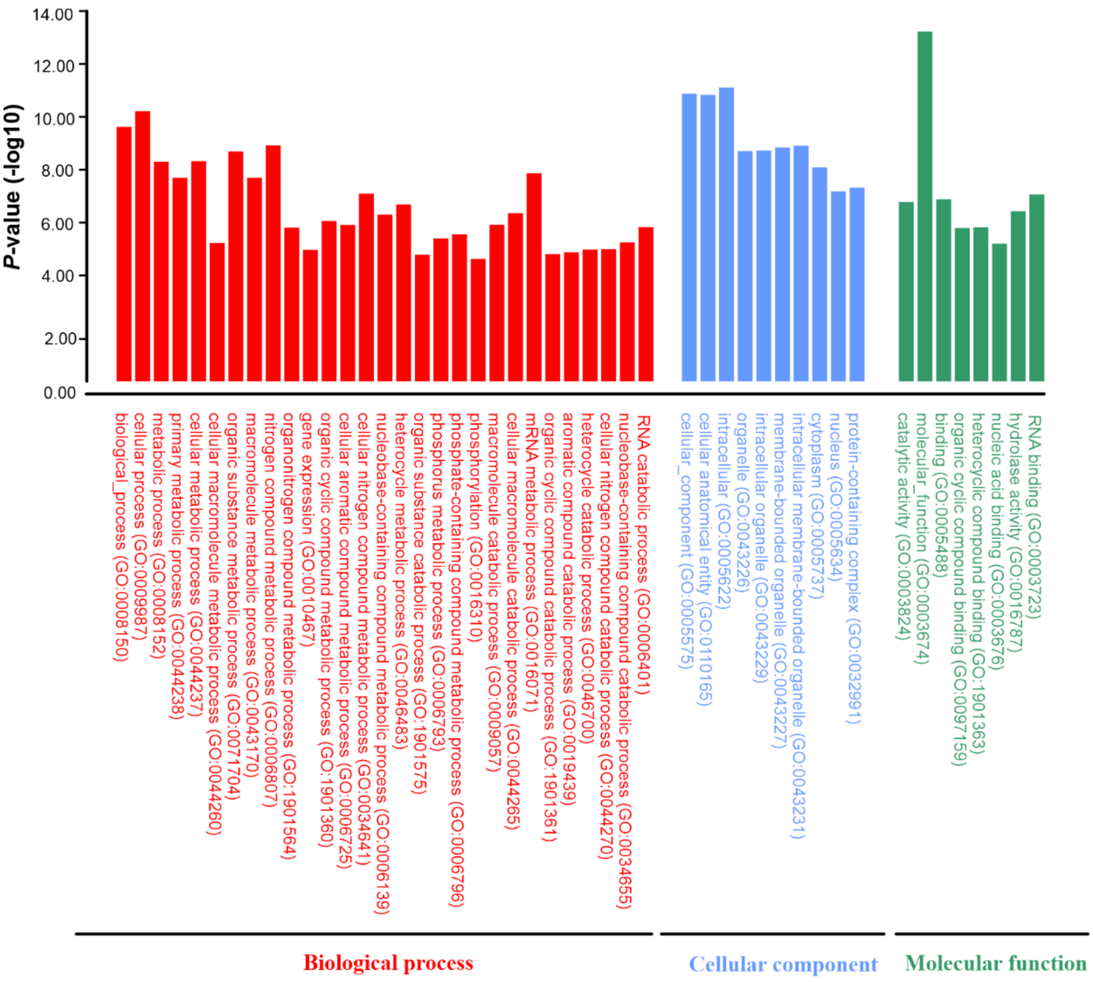

Figure S7. The enriched GO terms for co-expressed genes of four *SlHVA22* genes.

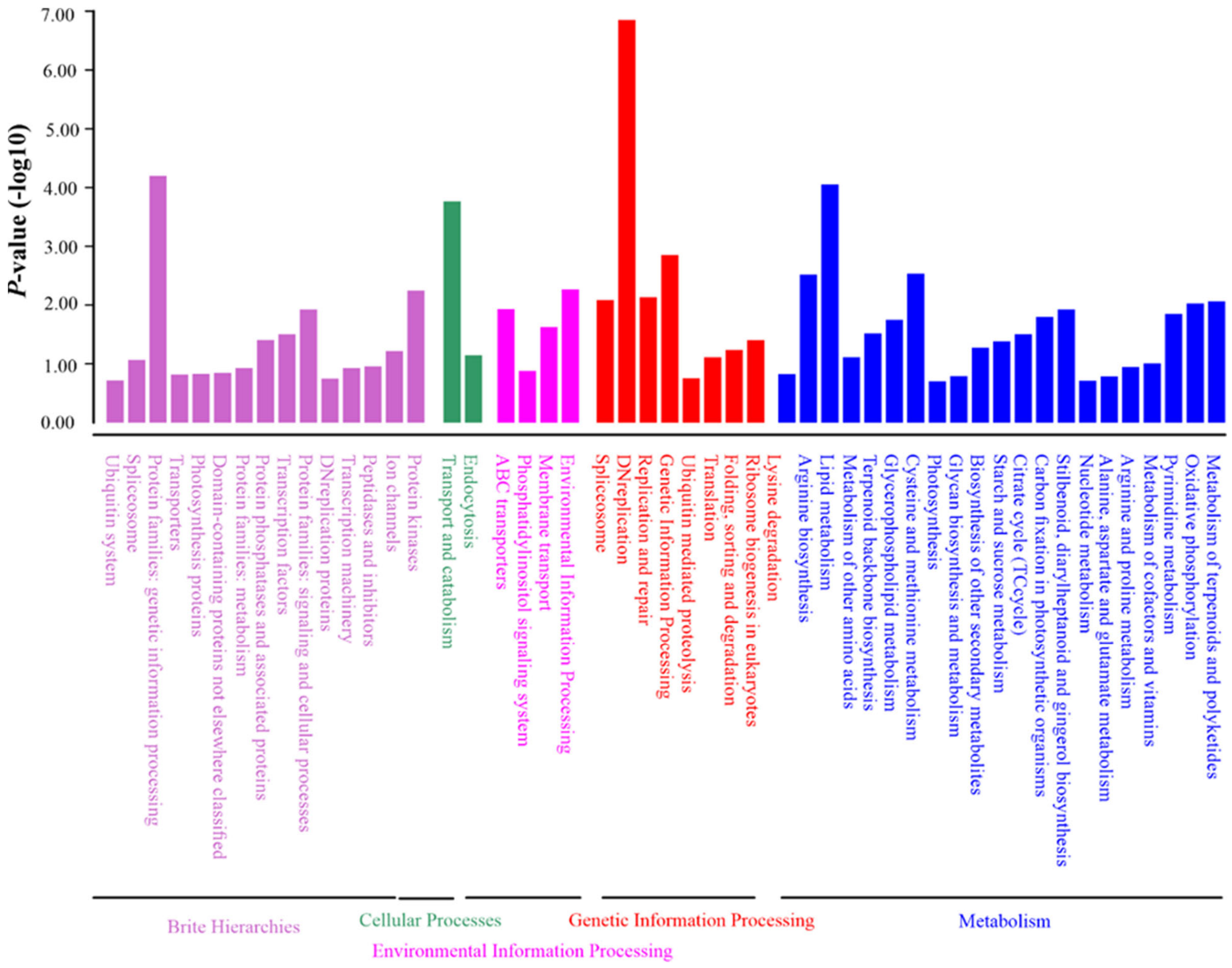

Figure S8. The biological pathways determined by KEGG analysis. The co-expressed genes of four *SlHVA22* genes having p-value less than 0.01 were subjected to KEGG analysis (see Figure 6).

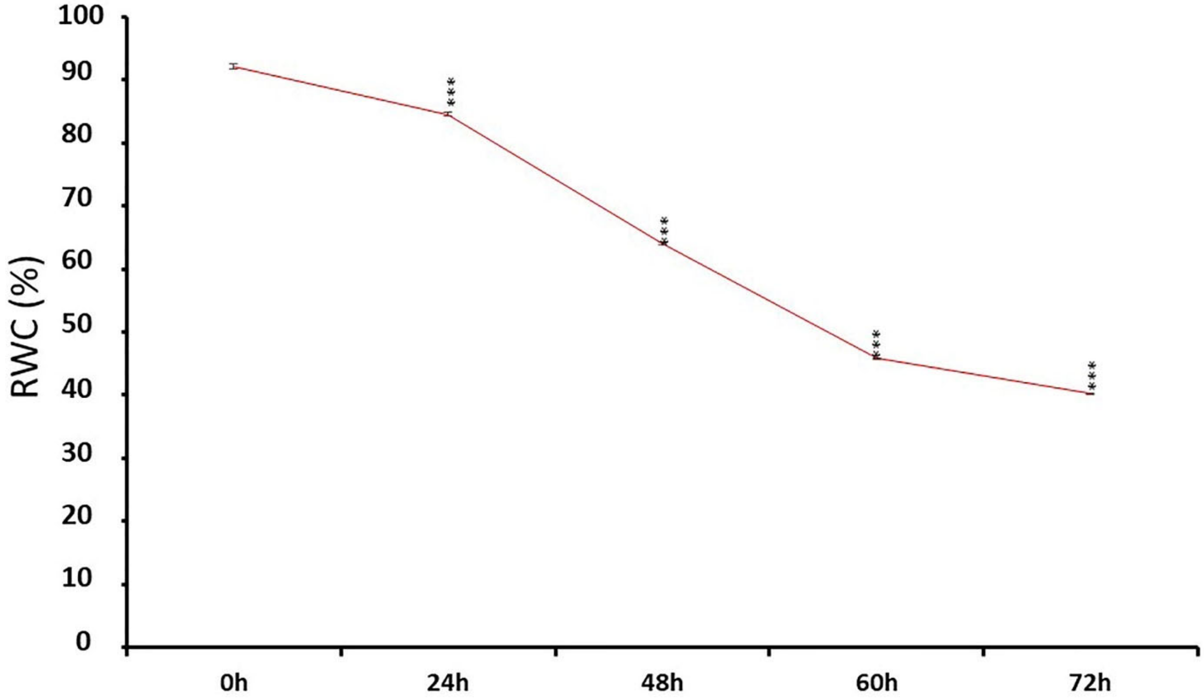

Figure S9. Measurement of relative water content (RWC) of leaf sampled at 0, 24, 48, 60 and 72h after the start of drought treatment. Error bars indicate standard deviations of the means of three independent biological replicates. The asterisk marks (* for p-value < 0.05, ** for p-value < 0.01, and *** for p-value < 0.001) analyzed using Student's *t*-tests denotes the significant differences between the samples (0h) and drought-treated samples.

Table S1. In silico analysis of the *HVA22* genes and corresponding proteins in tomato genome

Gene Name	Locus name	ORF (bp)	Chromosome No.	Protein					Subcellular Localization	No. of Introns
				Length (aa)	TB2/DP1/HVA22 domain Start-End (aa)	MW (kDa)	PI	GRAVY		
SlHVA22a	Solyc01g007780	663	1	220	19-97	25.80	9.35	-0.510	Nucleus	4
SlHVA22b	Solyc03g097420	411	3	136	24-98	15.84	9.37	0.210	Extracellular	4
SlHVA22c	Solyc03g110920	585	3	194	42-118	21.79	6.37	0.111	Endoplasmic reticulum	7
SlHVA22d	Solyc03g116350	531	3	176	32-107	20.50	6.41	0.005	Extracellular	4
SlHVA22e	Solyc04g014420	333	4	110	5-77	13.04	5.34	-0.034	Cytoplasm	2
SlHVA22f	Solyc04g076610	1410	4	469	32-108	53.34	9.03	-0.147	Nucleus	4
SlHVA22g	Solyc04g081340	915	4	304	19-96	33.64	8.95	-0.389	Chloroplast	4
SlHVA22h	Solyc05g007300	516	5	171	28-104	20.24	7.67	-0.101	Endoplasmic reticulum	4
SlHVA22i	Solyc06g072680	408	6	135	24-98	15.66	8.80	0.210	Chloroplast	4
SlHVA22j	Solyc10g007820	471	10	156	24-98	18.03	9.26	0.026	Chloroplast	4
SlHVA22k	Solyc10g047670	1680	10	559	24-100	63.67	8.73	-0.449	Nucleus	7
SlHVA22l	Solyc10g051300	564	10	187	32-108	21.86	7.02	-0.092	Endoplasmic reticulum	6
SlHVA22m	Solyc10g082040	570	10	189	19-97	22.23	8.84	-0.215	Extracellular	4
SlHVA22n	Solyc11g010930	429	11	142	24-98	16.85	8.58	0.065	Cytoplasm	2
SlHVA22o	Solyc12g089290	372	12	314	31-108	35.11	9.38	-0.399	Chloroplast	1

Table S2. Sequence homology across SlHVA22 proteins

	A	B	C	D	E	F	G	H	I	J	K	L	M	N	O
SlHVA22a (A)	100%														
SlHVA22b (B)	17.64%	100%													
SlHVA22c (C)	15.97%	21.32%	100%												
SlHVA22d (D)	13.63%	37.50%	18.18%	100%											
SlHVA22e (E)	16.36%	70.90%	22.72%	39.09%	100%										
SlHVA22f (F)	10.90%	27.20%	14.94%	27.84%	25.45%	100%									
SlHVA22g (G)	44.09%	21.32%	15.97%	17.61%	19.09%	12.50%	100%								
SlHVA22h (H)	16.37%	33.82%	22.80%	67.25%	37.27%	26.90%	21.05%	100%							
SlHVA22i (I)	17.77%	73.33%	22.22%	37.77%	69.09%	28.14%	19.25%	34.81%	100%						
SlHVA22j (J)	11.53%	44.11%	17.94%	44.87%	40.90%	27.56%	16.66%	44.23%	44.44%	100%					
SlHVA22k (K)	14.09%	33.08%	18.55%	37.50%	34.54%	11.72%	12.82%	39.18%	31.11%	32.05%	100%				
SlHVA22l (L)	16.04%	22.05%	60.96%	18.75%	23.63%	17.64%	18.71%	23.39%	22.96%	21.15%	21.39%	100%			
SlHVA22m (M)	63.49%	19.11%	19.57%	16.47%	17.27%	13.22%	49.20%	17.54%	18.51%	14.10%	15.87%	19.78%	100%		
SlHVA22n (N)	16.19%	71.32%	21.12%	36.61%	67.27%	23.23%	18.30%	31.69%	64.44%	39.43%	28.16%	21.12%	17.60%	100%	
SlHVA22o (O)	43.18%	22.05%	17.01%	17.61%	20%	14.01%	65.46%	19.88%	22.96%	15.38%	11.14%	19.25%	46.03%	21.12%	100%

Table S3. List of *cis*-acting elements in the upstream regions of *SlHVA22* genes

Gene	*Cis* element	Consensus sequence	Location	Strand	organism	Function
SlHVA22a	3-AF3 binding site	CACTATCTAAC	10	-	*Pisum sativum*	part of a conserved DNA module array (CMA3)
	GATA-motif	AAGATAAGATT	129	+	*Arabidopsis thaliana*	part of a light responsive element
	TGACG-motif	TGACG	157	+	*Hordeum vulgare*	cis-acting regulatory element involved in the MeJA-responsiveness
	TGA-box	TGACGTAA	157	+	*Glycine max*	part of an auxin-responsive element
	CGTCA-motif	CGTCA	157	-	*Hordeum vulgare*	cis-acting regulatory element involved in the MeJA-responsiveness
	CAAT-box	CAAAT	354	+	*Pisum sativum*	common cis-acting element in promoter and enhancer regions
	TCA-element	CCATCTTTTT	384	-	*Nicotiana tabacum*	cis-acting element involved in salicylic acid responsiveness
	TCT-motif	TCTTAC	406	-	*Arabidopsis thaliana*	part of a light responsive element
	ABRE	ACGTG	726	-	*Arabidopsis thaliana*	cis-acting element involved in the abscisic acid responsiveness
	G-Box	CACGTT	726	+	*Pisum*	cis-acting regulatory element involved in light

					sativum	responsiveness
	GT1-motif	GGTTAAT	890	+	*Avena sativa*	light responsive element
	Unnamed__1	GAATTTAATTAA	892	-	*Glycine max*	60K protein binding site
	Box 4	ATTAAT	895	-	*Petroselinum crispum*	part of a conserved DNA module involved in light responsiveness
	TCA-element	CCATCTTTTT	917	+	*Nicotiana tabacum*	cis-acting element involved in salicylic acid responsiveness
	AT-rich element	ATAGAAATCAA	1253	-	*Glycine max*	binding site of AT-rich DNA binding protein (ATBP-1)
SlHVA22b	TATC-box	TATCCCA	35	+	*Oryza sativa*	cis-acting element involved in gibberellin-responsiveness
	GCN4_motif	TGAGTCA	236	-	*Oryza sativa*	cis-regulatory element involved in endosperm expression
	Box 4	ATTAAT	257	+	*Petroselinum crispum*	part of a conserved DNA module involved in light responsiveness
	ABRE	CACGTG	322	+	*Arabidopsis thaliana*	cis-acting element involved in the abscisic acid responsiveness
	G-box	CACGTG	322	+	*Arabidopsis thaliana*	cis-acting regulatory element involved in light responsiveness
	Box 4	ATTAAT	374	+	*Petroselinum crispum*	part of a conserved DNA module involved in light responsiveness
	ARE	AAACCA	383	+	*Zea mays*	cis-acting regulatory element essential for the anaerobic induction
	O2-site	GATGA(C/T)(A/G)TG(A/G)	433	-	*Zea mays*	cis-acting regulatory element involved in zein

					metabolism regulation
Box 4	ATTAAT	488	+	*Petroselinum crispum*	part of a conserved DNA module involved in light responsiveness
CGTCA-motif	CGTCA	758	+	*Hordeum vulgare*	cis-acting regulatory element involved in the MeJA-responsiveness
CAT-box	GCCACT	939	-	*Arabidopsis thaliana*	cis-acting regulatory element related to meristem expression
ARE	AAACCA	1068	+	*Zea mays*	cis-acting regulatory element essential for the anaerobic induction
Box 4	ATTAAT	1139	-	*Petroselinum crispum*	part of a conserved DNA module involved in light responsiveness
ABRE	CACGTG	1149	-	*Arabidopsis thaliana*	cis-acting element involved in the abscisic acid responsiveness
G-box	CACGTG	1149	-	*Arabidopsis thaliana*	cis-acting regulatory element involved in light responsiveness
GT1-motif	GGTTAA	1179	-	*Arabidopsis thaliana*	light responsive element
MRE	AACCTAA	1181	+	*Petroselinum crispum*	MYB binding site involved in light responsiveness
3-AF3 binding site	CACTATCTAAC	1341	-	*Pisum sativum*	part of a conserved DNA module array (CMA3)
CGTCA-motif	CGTCA	1399	+	*Hordeum vulgare*	cis-acting regulatory element involved in the MeJA-responsiveness
O2-site	GTTGACGTGA	1401	-	*Zea mays*	cis-acting regulatory element involved in zein metabolism regulation

	ABRE	ACGTG	1402	-	*Arabidopsis thaliana*	cis-acting element involved in the abscisic acid responsiveness
	G-box	CACGTC	1402	+	*Zea mays*	cis-acting regulatory element involved in light responsiveness
	HD-Zip 1	CAAT(A/T)ATTG	1453	-	*Arabidopsis thaliana*	element involved in differentiation of the palisade mesophyll cells
SlHVA22c	TGACG-motif	TGACG	528	-	*Hordeum vulgare*	cis-acting regulatory element involved in the MeJA-responsiveness
	O2-site	GATGATGTGG	36	+	*Zea mays*	cis-acting regulatory element involved in zein metabolism regulation
	G-box	tgACACGTGGCA	125	+	*Lycopersicon esculentum*	cis-acting regulatory element involved in light responsiveness
	ABRE	GACACGTGGC	126	+	*Triticum aestivum*	cis-acting element involved in the abscisic acid responsiveness
	Unnamed__1	GAATTTAATTAA	165	+	*Glycine max*	60K protein binding site
	CGTCA-motif	CGTCA	528	+	*Hordeum vulgare*	cis-acting regulatory element involved in the MeJA-responsiveness
	Box 4	ATTAAT	752	-	*Petroselinum crispum*	part of a conserved DNA module involved in light responsiveness
	Box 4	ATTAAT	814	-	*Petroselinum crispum*	part of a conserved DNA module involved in light responsiveness
	G-box	ACACGTG(G/t)CACC	827	-	*Lycopersicon esculentum*	cis-acting regulatory element involved in light responsiveness
	ABRE	GCAACGTGTC	830	+	*Hordeum*	cis-acting element involved in the abscisic acid

					vulgare	responsiveness
	G-box	CACGTG	832	-	*Arabidopsis thaliana*	cis-acting regulatory element involved in light responsiveness
	ABRE	ACGTG	884	-	*Arabidopsis thaliana*	cis-acting element involved in the abscisic acid responsiveness
	GA-motif	ATAGATAA	925	+	*Arabidopsis thaliana*	part of a light responsive element
	Box 4	ATTAAT	939	-	*Petroselinum crispum*	part of a conserved DNA module involved in light responsiveness
	TCT-motif	TCTTAC	962	-	*Arabidopsis thaliana*	part of a light responsive element
	Box 4	ATTAAT	1028	-	*Petroselinum crispum*	part of a conserved DNA module involved in light responsiveness
	Box 4	ATTAAT	1191	-	*Petroselinum crispum*	part of a conserved DNA module involved in light responsiveness
	O2-site	GATGA(C/T)(A/G)TG(A/G)	1319	-	*Zea mays*	cis-acting regulatory element involved in zein metabolism regulation
	Box 4	ATTAAT	1377	-	*Petroselinum crispum*	part of a conserved DNA module involved in light responsiveness
	Box 4	ATTAAT	1487	-	*Petroselinum crispum*	part of a conserved DNA module involved in light responsiveness
SlHVA22d	TGACG-motif	TGACG	13	-	*Hordeum vulgare*	cis-acting regulatory element involved in the MeJA-responsiveness
	G-box	TACGTG	67	+	*Arabidopsis thaliana*	cis-acting regulatory element involved in light responsiveness

ABRE	ACGTG	68	+	*Arabidopsis thaliana*	cis-acting element involved in the abscisic acid responsiveness
TGACG-motif	TGACG	71	+	*Hordeum vulgare*	cis-acting regulatory element involved in the MeJA-responsiveness
ACE	CTAACGTATT	336	+	*Petroselinum crispum*	cis-acting element involved in light responsiveness
Gap-box	CAAATGAA(A/G)A	419	-	*Arabidopsis thaliana*	part of a light responsive element
MYB	CAACCA	542	-	*Arabidopsis thaliana*	involved in drought-inducibility
AT1-motif	AATTATTTTTTATT	806	-	*Solanum tuberosum*	part of a light responsive module
G-box	TACGTG	864	-	*Arabidopsis thaliana*	cis-acting regulatory element involved in light responsiveness
ABRE	ACGTG	864	-	*Arabidopsis thaliana*	cis-acting element involved in the abscisic acid responsiveness
Box 4	ATTAAT	877	-	*Petroselinum crispum*	part of a conserved DNA module involved in light responsiveness
chs-CMA1a	TTACTTAA	980	+	*Daucus carota*	part of a light responsive element
G-Box	CACGTT	1007	-	*Pisum sativum*	cis-acting regulatory element involved in light responsiveness
ABRE	ACGTG	1008	+	*Arabidopsis thaliana*	cis-acting element involved in the abscisic acid responsiveness
GATA-motif	AAGGATAAGG	1019	+	*Solanum*	part of a light responsive element

					tuberosum	
	ARE	AAACCA	1096	-	*Zea mays*	cis-acting regulatory element essential for the anaerobic induction
	TCA-element	CCATCTTTTT	1120	+	*Nicotiana tabacum*	cis-acting element involved in salicylic acid responsiveness
	Box III	atCATTTTCACt	1190	-	*Pisum sativum*	protein binding site
	GA-motif	ATAGATAA	1314	+	*Arabidopsis thaliana*	part of a light responsive element
	ATCT-motif	AATCTAATCC	1355	+	*Pisum sativum*	part of a conserved DNA module involved in light responsiveness
	Box 4	ATTAAT	1436	-	*Petroselinum crispum*	part of a conserved DNA module involved in light responsiveness
SlHVA22e	Box 4	ATTAAT	7	+	*Petroselinum crispum*	part of a conserved DNA module involved in light responsiveness
	G-box	TACGTG	42	-	*Arabidopsis thaliana*	cis-acting regulatory element involved in light responsiveness
	ABRE	ACGTG	42	-	*Arabidopsis thaliana*	cis-acting element involved in the abscisic acid responsiveness
	O2-site	GATGACATGG	118	+	*Zea mays*	cis-acting regulatory element involved in zein metabolism regulation
	circadian	CAAAGATATC	187	-	*Lycopersicon esculentum*	cis-acting regulatory element involved in circadian control
	I-box	cCATATCCAAT	199	+	*Flaveria trinervia*	part of a light responsive element

	Box 4	ATTAAT	254	+	*Petroselinum crispum*	part of a conserved DNA module involved in light responsiveness
	GT1-motif	GGTTAA	460	-	*Arabidopsis thaliana*	light responsive element
	GT1-motif	GGTTAAT	635	+	*Avena sativa*	light responsive element
	MBS	CAACTG	656	-	*Arabidopsis thaliana*	MYB binding site involved in drought-inducibility
	LAMP-element	CTTTATCA	750	+	*Pisum sativum*	part of a light responsive element
	AE-box	AGAAACTT	806	-	*Arabidopsis thaliana*	part of a module for light response
	O2-site	GATGATGTGG	1007	+	*Zea mays*	cis-acting regulatory element involved in zein metabolism regulation
	Box 4	ATTAAT	1053	-	*Petroselinum crispum*	part of a conserved DNA module involved in light responsiveness
	Box II	TGGTAATAA	1244	+	*Solanum tuberosum*	part of a light responsive element
SlHVA22f	TGA-element	AACGAC	124	-	*Brassica oleracea*	auxin-responsive element
	AE-box	AGAAACAA	151	-	*Arabidopsis thaliana*	part of a module for light response
	G-box	CACGTC	372	-	*Zea mays*	cis-acting regulatory element involved in light responsiveness
	ABRE	ACGTG	373	+	*Arabidopsis*	cis-acting element involved in the abscisic acid

					thaliana	responsiveness
	G-box	CACGAC	403	+	*Zea mays*	cis-acting regulatory element involved in light responsiveness
	TGA-element	AACGAC	931	+	*Brassica oleracea*	auxin-responsive element
	Box 4	ATTAAT	945	-	*Petroselinum crispum*	part of a conserved DNA module involved in light responsiveness
	GT1-motif	GGTTAA	962	-	*Arabidopsis thaliana*	light responsive element
	HD-Zip 1	CAAT(A/T)ATTG	1102	-	*Arabidopsis thaliana*	element involved in differentiation of the palisade mesophyll cells
	Box 4	ATTAAT	1181	-	*Petroselinum crispum*	part of a conserved DNA module involved in light responsiveness
	ARE	AAACCA	1246	+	*Zea mays*	cis-acting regulatory element essential for the anaerobic induction
	ARE	AAACCA	1401	+	*Zea mays*	cis-acting regulatory element essential for the anaerobic induction
	RY-element	CATGCATG	1418	-	*Helianthus annuus*	cis-acting regulatory element involved in seed-specific regulation
	ARE	AAACCA	1461	-	*Zea mays*	cis-acting regulatory element essential for the anaerobic induction
SlHVA22g	CGTCA-motif	CGTCA	64	-	*Hordeum vulgare*	cis-acting regulatory element involved in the MeJA-responsiveness
	A-box	CCGTCC	149	+	*Petroselinum crispum*	cis-acting regulatory element

TCA-element	CCATCTTTTT	153	+	*Nicotiana tabacum*	cis-acting element involved in salicylic acid responsiveness
AT1-motif	AATTATTTTTTATT	172	-	*Solanum tuberosum*	part of a light responsive module
ARE	AAACCA	492	-	*Zea mays*	cis-acting regulatory element essential for the anaerobic induction
TGA-element	AACGAC	510	+	*Brassica oleracea*	auxin-responsive element
GC-motif	CCCCCG	527	+	*Zea mays*	enhancer-like element involved in anoxic specific inducibility
GT1-motif	GTGTGTGAA	542	-	*Solanum tuberosum*	light responsive element
CCAAT-box	CAACGG	569	+	*Hordeum vulgare*	MYBHv1 binding site
MSA-like	(T/C)C(T/C)AACGG(T/C)(T/C)A	641	+	*Catharanthus roseus*	cis-acting element involved in cell cycle regulation
P-box	CCTTTTG	703	-	*Oryza sativa*	gibberellin-responsive element
LAMP-element	CTTTATCA	916	-	*Pisum sativum*	part of a light responsive element
TCA-element	CCATCTTTTT	935	-	*Nicotiana tabacum*	cis-acting element involved in salicylic acid responsiveness
GARE-motif	TCTGTTG	1083	-	*Brassica oleracea*	gibberellin-responsive element
MYB	CAACCA	1038	-	*Arabidopsis*	involved in drought-inducibility

					thaliana	
	ATC-motif	AGCTATCCA	1151	-	*Arabidopsis thaliana*	part of a conserved DNA module involved in light responsiveness
	ATC-motif	AGTAATCT	1166	+	*Spinacia oleracea*	part of a conserved DNA module involved in light responsiveness
	ARE	AAACCA	1183	-	*Zea mays*	cis-acting regulatory element essential for the anaerobic induction
	ARE	AAACCA	1272	-	*Zea mays*	cis-acting regulatory element essential for the anaerobic induction
	ARE	AAACCA	1450	-	*Zea mays*	cis-acting regulatory element essential for the anaerobic induction
SlHVA22h	TGACG-motif	TGACG	123	+	*Hordeum vulgare*	cis-acting regulatory element involved in the MeJA-responsiveness
	3-AF1 binding site	TAAGAGAGGAA	200	+	*Solanum tuberosum*	light responsive element
	ATCT-motif	AATCTAATCC	285	-	*Pisum sativum*	part of a conserved DNA module involved in light responsiveness
	ACE	CTAACGTATT	313	-	*Petroselinum crispum*	cis-acting element involved in light responsiveness
	Box 4	ATTAAT	378	+	*Petroselinum crispum*	part of a conserved DNA module involved in light responsiveness
	Myb-binding site	CAACAG	531	-	*Nicotiana tabacum*	involved in drought-inducibility
	Myb-binding site	CAACAG	663	-	*Nicotiana tabacum*	involved in drought-inducibility

MRE	AACCTAA	638	-	*Petroselinum crispum*	MYB binding site involved in light responsiveness
P-box	CCTTTTG	673	-	*Oryza sativa*	gibberellin-responsive element
G-Box	CACGTT	818	-	*Pisum sativum*	cis-acting regulatory element involved in light responsiveness
ABRE	ACGTG	819	+	*Arabidopsis thaliana*	cis-acting element involved in the abscisic acid responsiveness
G-box	CACGTC	830	-	*Zea mays*	cis-acting regulatory element involved in light responsiveness
ABRE	ACGTG	831	+	*Arabidopsis thaliana*	cis-acting element involved in the abscisic acid responsiveness
GT1-motif	GGTTAA	842	+	*Arabidopsis thaliana*	light responsive element
4cl-CMA2b	TCTCACCAACCACA	860	+	*Solanum tuberosum*	light responsive element
Box 4	ATTAAT	984	-	*Petroselinum crispum*	part of a conserved DNA module involved in light responsiveness
LTR	CCGAAA	1045	+	*Hordeum vulgare*	cis-acting element involved in low-temperature responsiveness
P-box	CCTTTTG	1065	-	*Oryza sativa*	gibberellin-responsive element
AE-box	AGAAACTT	1155	-	*Arabidopsis thaliana*	part of a module for light response
TGACG-motif	TGACG	1217	+	*Hordeum vulgare*	cis-acting regulatory element involved in the MeJA-responsiveness

	3-AF3 binding site	CACTATCTAAC	1245	+	*Pisum sativum*	part of a conserved DNA module array (CMA3)
	LTR	CCGAAA	1358	-	*Hordeum vulgare*	cis-acting element involved in low-temperature responsiveness
SlHVA22i	AE-box	AGAAACTT	4	+	*Arabidopsis thaliana*	part of a module for light response
	MRE	AACCTAA	78	-	*Petroselinum crispum*	MYB binding site involved in light responsiveness
	Box 4	ATTAAT	142	+	*Petroselinum crispum*	part of a conserved DNA module involved in light responsiveness
	TGA-element	AACGAC	245	-	*Brassica oleracea*	auxin-responsive element
	3-AF1 binding site	TAAGAGAGGAA	284	-	*Solanum tuberosum*	light responsive element
	TGACG-motif	TGACG	724	-	*Hordeum vulgare*	cis-acting regulatory element involved in the MeJA-responsiveness
	AT-rich element	ATAGAAATCAA	793	+	*Glycine max*	binding site of AT-rich DNA binding protein (ATBP-1)
	O2-site	GATGATGTGG	1055	-	*Zea mays*	cis-acting regulatory element involved in zein metabolism regulation
	CGTCA-motif	CGTCA	1284	+	*Hordeum vulgare*	cis-acting regulatory element involved in the MeJA-responsiveness
	MBS	CAACTG	1312	+	*Arabidopsis thaliana*	MYB binding site involved in drought-inducibility
	ABRE	ACGTG	1367	-	*Arabidopsis*	cis-acting element involved in the abscisic acid

					thaliana	responsiveness
	G-box	CACGTC	1367	+	*Zea mays*	cis-acting regulatory element involved in light responsiveness
	G-Box	CACGTG	1415	-	*Pisum sativum*	cis-acting regulatory element involved in light responsiveness
	ABRE	ACGTG	1416	+	*Arabidopsis thaliana*	cis-acting element involved in the abscisic acid responsiveness
SlHVA22j	TCT-motif	TCTTAC	17	-	*Arabidopsis thaliana*	part of a light responsive element
	AE-box	AGAAACTT	26	+	*Arabidopsis thaliana*	part of a module for light response
	GT1-motif	GTGTGTGAA	103	+	*Solanum tuberosum*	light responsive element
	AE-box	AGAAACAA	217	+	*Arabidopsis thaliana*	part of a module for light response
	ARE	AAACCA	271	-	*Zea mays*	cis-acting regulatory element essential for the anaerobic induction
	TCT-motif	TCTTAC	343	+	*Arabidopsis thaliana*	part of a light responsive element
	TCT-motif	TCTTAC	515	+	*Arabidopsis thaliana*	part of a light responsive element
	ATCT-motif	AATCTAATCC	575	+	*Pisum sativum*	part of a conserved DNA module involved in light responsiveness
	G-Box	CACGTG	590	+	*Pisum*	cis-acting regulatory element involved in light

				sativum	responsiveness
ABRE	ACGTG	591	+	*Arabidopsis thaliana*	cis-acting element involved in the abscisic acid responsiveness
TCT-motif	TCTTAC	711	-	*Arabidopsis thaliana*	part of a light responsive element
GT1-motif	GGTTAAT	761	-	*Avena sativa*	light responsive element
GT1-motif	GGTTAA	762	-	*Arabidopsis thaliana*	light responsive element
MYB	CAACCA	968	-	Arabidopsis thaliana	involved in drought-inducibility
O2-site	GTTGACGTGA	970	+	*Zea mays*	cis-acting regulatory element involved in zein metabolism regulation
TGACG-motif	TGACG	972	+	*Hordeum vulgare*	cis-acting regulatory element involved in the MeJA-responsiveness
G-box	CACGTC	973	-	*Zea mays*	cis-acting regulatory element involved in light responsiveness
ABRE	ACGTG	974	+	*Arabidopsis thaliana*	cis-acting element involved in the abscisic acid responsiveness
RY-element	CATGCATG	1035	-	*Helianthus annuus*	cis-acting regulatory element involved in seed-specific regulation
TC-rich repeats	GTTTTCTTAC	1174	-	*Nicotiana tabacum*	cis-acting element involved in defense and stress responsiveness
TCT-motif	TCTTAC	1174	-	*Arabidopsis thaliana*	part of a light responsive element

	MYB	CAACCA	1244	-	Arabidopsis thaliana	involved in drought-inducibility
	GARE-motif	TCTGTTG	1244	-	*Brassica oleracea*	gibberellin-responsive element
	Box 4	ATTAAT	1424	-	*Petroselinum crispum*	part of a conserved DNA module involved in light responsiveness
SlHVA22k	P-box	CCTTTTG	30	-	*Oryza sativa*	gibberellin-responsive element
	CGTCA-motif	CGTCA	211	+	*Hordeum vulgare*	cis-acting regulatory element involved in the MeJA-responsiveness
	MYB	CAACCA	226	+	*Arabidopsis thaliana*	involved in drought-inducibility
	O2-site	GATGATGTGG	229	-	*Zea mays*	cis-acting regulatory element involved in zein metabolism regulation
	ABRE	ACGTG	280	-	*Arabidopsis thaliana*	cis-acting element involved in the abscisic acid responsiveness
	G-box	TACGTG	280	-	*Arabidopsis thaliana*	cis-acting regulatory element involved in light responsiveness
	MYB	CAACCA	656	-	*Arabidopsis thaliana*	involved in drought-inducibility
	GA-motif	ATAGATAA	1027	+	*Arabidopsis thaliana*	part of a light responsive element
	ARE	AAACCA	1171	+	*Zea mays*	cis-acting regulatory element essential for the anaerobic induction
	Box 4	ATTAAT	1195	-	*Petroselinum*	part of a conserved DNA module involved in light

					crispum	responsiveness
	GCN4_motif	TGAGTCA	1209	-	*Oryza sativa*	cis-regulatory element involved in endosperm expression
	Box 4	ATTAAT	1379	-	*Petroselinum crispum*	part of a conserved DNA module involved in light responsiveness
	TCA-element	TCAGAAGAGG	1428	-	*Brassica oleracea*	cis-acting element involved in salicylic acid responsiveness
	MBS	CAACTG	1469	-	*Arabidopsis thaliana*	MYB binding site involved in drought-inducibility
SlHVA22l	G-Box	CACGTT	3	-	*Pisum sativum*	cis-acting regulatory element involved in light responsiveness
	ABRE	ACGTG	4	+	*Arabidopsis thaliana*	cis-acting element involved in the abscisic acid responsiveness
	GATA-motif	GATAGGG	134	-	*Pisum sativum*	part of a light responsive element
	MYB	CAACCA	270	-	*Arabidopsis thaliana*	involved in drought-inducibility
	GATA-motif	AAGATAAGATT	295	+	*Arabidopsis thaliana*	part of a light responsive element
	TCT-motif	TCTTAC	485	+	*Arabidopsis thaliana*	part of a light responsive element
	MBS	CAACTG	923	+	*Arabidopsis thaliana*	MYB binding site involved in drought-inducibility
	Gap-box	CAAATGAA(A/G)A	1129	+	*Arabidopsis thaliana*	part of a light responsive element

	Box 4	ATTAAT	1222	-	*Petroselinum crispum*	part of a conserved DNA module involved in light responsiveness
	GT1-motif	GGTTAA	1270	-	*Arabidopsis thaliana*	light responsive element
	GT1-motif	GGTTAA	1344	+	*Arabidopsis thaliana*	light responsive element
	MBS	CAACTG	1479	-	*Arabidopsis thaliana*	MYB binding site involved in drought-inducibility
SlHVA22m	LTR	CCGAAA	1	-	*Hordeum vulgare*	cis-acting element involved in low-temperature responsiveness
	Box 4	ATTAAT	30	+	*Petroselinum crispum*	part of a conserved DNA module involved in light responsiveness
	ARE	AAACCA	39	-	*Zea mays*	cis-acting regulatory element essential for the anaerobic induction
	Box 4	ATTAAT	58	+	*Petroselinum crispum*	part of a conserved DNA module involved in light responsiveness
	Box 4	ATTAAT	70	+	*Petroselinum crispum*	part of a conserved DNA module involved in light responsiveness
	Box 4	ATTAAT	97	+	*Petroselinum crispum*	part of a conserved DNA module involved in light responsiveness
	CGTCA-motif	CGTCA	183	-	*Hordeum vulgare*	cis-acting regulatory element involved in the MeJA-responsiveness
	AE-box	AGAAACAA	228	+	*Arabidopsis thaliana*	part of a module for light response

TCT-motif	TCTTAC	296	+	*Arabidopsis thaliana*	part of a light responsive element
G-Box	CACGTT	355	-	*Pisum sativum*	cis-acting regulatory element involved in light responsiveness
ABRE	ACGTG	356	+	*Arabidopsis thaliana*	cis-acting element involved in the abscisic acid responsiveness
GT1-motif	GGTTAA	375	+	*Arabidopsis thaliana*	light responsive element
Box 4	ATTAAT	449	+	*Petroselinum crispum*	part of a conserved DNA module involved in light responsiveness
ARE	AAACCA	485	+	*Zea mays*	cis-acting regulatory element essential for the anaerobic induction
Box 4	ATTAAT	492	+	*Petroselinum crispum*	part of a conserved DNA module involved in light responsiveness
MYB	CAACCA	611	-	*Arabidopsis thaliana*	involved in drought-inducibility
G-box	CACGTC	653	+	*Zea mays*	cis-acting regulatory element involved in light responsiveness
ABRE	ACGTG	653	-	*Arabidopsis thaliana*	cis-acting element involved in the abscisic acid responsiveness
TGACG-motif	TGACG	766	+	*Hordeum vulgare*	cis-acting regulatory element involved in the MeJA-responsiveness
CGTCA-motif	CGTCA	766	-	*Hordeum vulgare*	cis-acting regulatory element involved in the MeJA-responsiveness
GATA-motif	AAGATAAGATT	822	-	*Arabidopsis*	part of a light responsive element

				thaliana	
G-box	TACGTG	842	+	*Arabidopsis thaliana*	cis-acting regulatory element involved in light responsiveness
ABRE	ACGTG	843	+	*Arabidopsis thaliana*	cis-acting element involved in the abscisic acid responsiveness
AT-rich sequence	TAAAATACT	853	+	*Pisum sativum*	element for maximal elicitor-mediated activation (2copies)
CCAAT-box	CAACGG	935	-	*Hordeum vulgare*	MYBHv1 binding site
Box 4	ATTAAT	976	-	*Petroselinum crispum*	part of a conserved DNA module involved in light responsiveness
Box 4	ATTAAT	1042	-	*Petroselinum crispum*	part of a conserved DNA module involved in light responsiveness
Box 4	ATTAAT	1132	-	*Petroselinum crispum*	part of a conserved DNA module involved in light responsiveness
Box 4	ATTAAT	1158	-	*Petroselinum crispum*	part of a conserved DNA module involved in light responsiveness
TCT-motif	TCTTAC	1219	-	*Arabidopsis thaliana*	part of a light responsive element
ABRE	ACGTG	1233	-	*Arabidopsis thaliana*	cis-acting element involved in the abscisic acid responsiveness
G-Box	CACGTT	1233	+	*Pisum sativum*	cis-acting regulatory element involved in light responsiveness
Box 4	ATTAAT	1385	-	*Petroselinum*	part of a conserved DNA module involved in light

					crispum	responsiveness
SlHVA22n	GT1-motif	GGTTAA	2	+	*Arabidopsis thaliana*	light responsive element
	Box 4	ATTAAT	8	+	*Petroselinum crispum*	part of a conserved DNA module involved in light responsiveness
	MYB	CAACCA	70	-	*Arabidopsis thaliana*	involved in drought-inducibility
	O2-site	GTTGACGTGA	184	-	*Zea mays*	cis-acting regulatory element involved in zein metabolism regulation
	LS7	CAGATTTATTTTTA	296	-	*Zea mays*	part of a light responsive element
	Box 4	ATTAAT	324	+	*Petroselinum crispum*	part of a conserved DNA module involved in light responsiveness
	ABRE	GACACGTGGC	450	+	*Triticum aestivum*	cis-acting element involved in the abscisic acid responsiveness
	G-Box	CACGTG	452	+	*Pisum sativum*	cis-acting regulatory element involved in light responsiveness
	Box 4	ATTAAT	528	+	*Petroselinum crispum*	part of a conserved DNA module involved in light responsiveness
	TCT-motif	TCTTAC	580	+	*Arabidopsis thaliana*	part of a light responsive element
	MBS	CAACTG	899	-	*Arabidopsis thaliana*	MYB binding site involved in drought-inducibility
	Box 4	ATTAAT	1000	-	*Petroselinum crispum*	part of a conserved DNA module involved in light responsiveness

	TC-rich repeats	GTTTTCTTAC	1486	+	*Nicotiana tabacum*	cis-acting element involved in defense and stress responsiveness
	TCT-motif	TCTTAC	1490	+	*Arabidopsis thaliana*	part of a light responsive element
SlHVA22o	ACA-motif	AATCACAACCATA	9	-	*Arabidopsis thaliana*	part of gapA in (gapA-CMA1) involved with light responsiveness
	Box 4	ATTAAT	80	+	*Petroselinum crispum*	part of a conserved DNA module involved in light responsiveness
	Box 4	ATTAAT	112	+	*Petroselinum crispum*	part of a conserved DNA module involved in light responsiveness
	Box 4	ATTAAT	177	+	*Petroselinum crispum*	part of a conserved DNA module involved in light responsiveness
	CGTCA-motif	CGTCA	319	-	*Hordeum vulgare*	cis-acting regulatory element involved in the MeJA-responsiveness
	Box 4	ATTAAT	412	+	*Petroselinum crispum*	part of a conserved DNA module involved in light responsiveness
	Box 4	ATTAAT	421	+	*Petroselinum crispum*	part of a conserved DNA module involved in light responsiveness
	ACE	CTAACGTATT	427	-	*Petroselinum crispum*	cis-acting element involved in light responsiveness
	MYB	CAACCA	495	-	*Arabidopsis thaliana*	involved in drought-inducibility
	LAMP-element	CCTTATCCA	604	-	*Spinacia oleracea*	part of a light responsive element

Box 4	ATTAAT	707	+	*Petroselinum crispum*	part of a conserved DNA module involved in light responsiveness
Sp1	GGGCGG	818	+	*Oryza sativa*	light responsive element
P-box	CCTTTTG	829	+	*Oryza sativa*	gibberellin-responsive element
GATA-motif	GATAGGG	843	-	*Pisum sativum*	part of a light responsive element
GT1-motif	GGTTAA	1066	-	*Arabidopsis thaliana*	light responsive element
MYB	CAACCA	1071	-	*Arabidopsis thaliana*	involved in drought-inducibility
MBSI	TTTTTACGGTTA	1091	+	*Petunia hybrida*	MYB binding site involved in flavonoid biosynthetic genes regulation
CGTCA-motif	CGTCA	1280	+	*Hordeum vulgare*	cis-acting regulatory element involved in the MeJA-responsiveness

Table S4. miRNA target sites predicted in tomato *HVA22* genes

**MIRNA_Acc.**	**Target_Acc.**	**Expectation**	**UPE$**	**MIRNA_start**	**MIRNA_end**	**Target_start**	**Target_end**	**MIRNA_aligned_fragment**	**alignment**	**Target_aligned_fragment**	**Inhibition**
sly-MIR9474-5p	*SlHVA22a*	5	-1	1	22	1045	1066	UGUAGAAGUCAUGAAUAAAAUG	: :::: :.:::..:: ::::	CCUUUUUCUUAUGGUUUAUACA	Cleavage

Sly-MIR5303a	*SlHVA22b*	5	-1	1	21	586	606	UUUUUGAAGAG UUCGAGCAAC	::.:. :::::::: :::	UGUGUUUACAC UCUUCACAAA	Cleavage
sly-MIR5303	*SlHVA22b*	5	-1	1	21	586	606	UUUUUGAAGAG UUCGAGCAAC	::.:. :::::::: :::	UGUGUUUACAC UCUUCACAAA	Cleavage
Sly-MIR9479	*SlHVA22c*	5	-1	1	22	825	846	GAGAAUGGUAG AGGGUCGGACC	::: :.:::::...: ::.	GUACCGUCUCU CUAUUGUACUU	Cleavage
sly-MIR9475-5p	*SlHVA22c*	4	-1	1	21	314	334	AACGAUCUCUA CAUUGUAGGC	::.. :::::.:::.: ::	UGCUGUUCUGU AGGGAUUGUU	Cleavage
sly-MIR9479-3p	*SlHVA22c*	5	-1	1	22	825	846	GAGAAUGGUAG AGGGUCGGACC	::: :.:::::...: ::.	GUACCGUCUCU CUAUUGUACUU	Cleavage
Sly-MIR395a	*SlHVA22d*	5	-1	1	21	904	924	CUGAAGUGUUU GGGGGAACUC	:::.::.:.:. ::::..	CUUUUCUCCUA GAUACUUUGU	Cleavage
Sly-MIR396a	*SlHVA22d*	5	-1	1	21	737	757	UUCCACAGCUU UCUUGAACUG	: :::.:.:::.: : : :::.	CUGUUUAGGAA GGCAGAGGAG	Cleavage
sly-MIR166c-5p	*SlHVA22d*	4.5	-1	1	21	576	596	GGGAUGUUGUC UGGCUCGACA	:::::::..:: ::::.	UAGUAAGCCAG AUGACAUCCU	Cleavage

sly-<br>MIR39<br>6a-5p	*SlHV*<br>*A22d*	5	-1	1	21	737	757	UUCCACAGCUU<br>UCUUGAACUG	:<br>:::.:.:::.:<br>: : :::.	CUGUUUAGGAA<br>GGCAGAGGAG	Clea<br>vage
sly-<br>MIR16<br>6c-5p	*SlHV*<br>*A22f*	4	-1	1	21	877	897	GGGAUGUUGUC<br>UGGCUCGACA	:.::<br>:::::.::<br>:::.:	GUUUGAACCAG<br>AUAAAAUCUC	Clea<br>vage
sly-<br>MIR19<br>17	*SlHV*<br>*A22g*	5	-1	1	21	1302	1322	AUUAAUAAAGA<br>GUGCUAAAGU	:::<br>::.::<br>:::::::	CAAUUACCAUU<br>CAUUAUUAAA	Clea<br>vage
Sly-<br>MIR48<br>2a	*SlHV*<br>*A22j*	5	-1	1	22	47	68	UUUCCAAUUCC<br>ACCCAUUCCUA	::::::::<br>:::::::	UUCCAAUGGGU<br>GCUAUUGGAAC	Tran<br>slatio<br>n
Sly-<br>MIR60<br>24	*SlHV*<br>*A22j*	5	-1	1	21	58	78	UUUAGCAAGAG<br>UUGUUUUACC	: :: .<br>::::<br>::::::::	GCUAUUGGAAC<br>UAUUGCUAAA	Clea<br>vage
Sly-<br>MIR10<br>535	*SlHV*<br>*A22k*	4	-1	1	24	301	323	UUGGCAUAAGU<br>UUGUGAAAGCC<br>GG	:::<br>::::::::::<br>::::	ACCUAUUUGAC<br>AAACUUAU-<br>CCAA	Clea<br>vage
Sly-<br>MIR15<br>9b	*SlHV*<br>*A22k*	4	-1	1	20	101	120	AUUUACUCUUC<br>UAGGAUCCU	:: :.<br>:::::::::::<br>:	UUGAACUGAGA<br>AGAGUAAAU	Clea<br>vage
Sly-<br>MIR60<br>23	*SlHV*<br>*A22k*	4.5	-1	1	22	576	597	UUACUACGGUC<br>AGGAAUCUUAU	::<br>:::.:::<br>:.::.:::.	GAAACAUUUCU<br>GUCUGUGGUAG	Tran<br>slatio<br>n

sly-MIR9475-5p	*SlHVA22l*	5	-1	1	21	271	291	AACGAUCUCUACAUUGUAGGC	::..<br>:::.:.:.:.:<br>::	UGCUGUUCUGUGGGGGUUGUU	Cleavage
Sly-MIR395a	*SlHVA22m*	5	-1	1	21	464	484	CUGAAGUGUUUGGGGGAACUC	:::::..<br>::::..:::<br>::	AAGUUCUUGCAAAUGCUUGAG	Cleavage
sly-MIR5302a	*SlHVA22n*	4.5	-1	1	21	481	501	AAACGAGGUUUGUUACUUUGG	:.:<br>::::::...:.<br>:::	GAAGAUUAACAAGUUUUGUUG	Cleavage
Sly-MIR6024	*SlHVA22o*	5	-1	1	21	707	727	UUUAGCAAGAGUUGUUUUACC	:::::.::::<br>:::.::	CAAAAAACGACUCCAGCUGAA	Cleavage
sly-MIR9470-5p	*SlHVA22o*	4.5	-1	1	21	211	231	UGAAAUCCAUGAGCCUAAACU	::: :<br>.::::::.::<br>::	GCUUUUGUUUCAUGGGUUCCA	Cleavage

Table S5. Target sites for post-translational modifications of tomato HVA22 proteins

Protein	Phosphorylation sites	N-glycosylation sites
SlHVA22a	S9, S30, S55, T86, T92, S99, S143, S145, S149, S153, S162, S165, S169, S173, S175, S176, S177, S178, S179, S186, S189, S190, S199, S202, S209, S212, S214, S215	N126
SlHVA22b	T9, S28, T35, S36, T54, Y105, S107, S108, Y109, S114, S116, T121, T130	
SlHVA22c	S12, S22, S36, T46, T52, S70, S72, Y83, Y111, T112, S161, T177, T187, S188, S190, S194	N10
SlHVA22d	S17, Y34, S36, T42, S44, T62, S79, Y104, T107, S128, T135, Y140, T146, S165, Y174	
SlHVA22e	S7, S13, T14, S15, T33, T69, Y72. T74, Y85, S90, T100, Y109	
SlHVA22f	S5, S16, S26, Y34, S36, S42, S46, S47, Y48, T67, S76, S86, S110, T119, S124, S130, S135, S139, T150, S154, T160, S166, S173, Y175, Y186, S201, S202, T208, S229, S258, S265, S278, S284, T305, S321, T349, S380, S401, S421, T425, Y431, S433, S438, S464,	
SlHVA22g	S4, Y21, T51, T86, Y88, S108, S129, S144, S146, T152, T154, S158, S159, T165, S167, S172, S173, S175, S189, S190, S192, S193, S194, S200, S206, S207, T223, T224, S226, S228, S231, T232, S236, T239, S243, S245, S247, S255, T256, S257, S271, S282, S295, T298, S301	
SlHVA22h	S3, S24, Y30, T31, S32, T38, S40, T49, S55, T58, S63, S65, S75, S123, T130, Y135, T157, T164, Y169	
SlHVA22i	T30, S18, T35, S36, T54, Y105, S114, S121, S126, T129, S130, T134	
SlHVA22j	T6, S27, S28, S34, S36, T45, S51, T54, S123, S129, Y133	
SlHVA22k	S7, S28, T34, S54, S108, S110, S115, Y117, S128, S146, S154, S165, Y176, Y179, S180, S181, T191, T193, S194, T201, T205, S210, T218, S236, T249, T278, T289, T290, S297, S306, Y308, T317, S320, S323, S349, S361, T373, T374, T378, T381, S397, T413, T415, S416, T427, T440, T442, S448, S471, T472, T485, T493, S510, S540, T543, Y550	N113, N163, N203, N321
SlHVA22l	S12, S26, T36, S62, Y73, T103, S132, S168, T180, S181, S83	
SlHVA22m	S55, S62, S87, T92, T103, S108, Y141, S144, S146, T150, S155, T161, T162, S166, S167, Y176, S185	N126
SlHVA22n	S28, T34, S35, S51, T54, S108, S109, S110, S111, S112, S119, S1124, T133, Y142	
SlHVA22o	S16, Y33, T63, T98, Y100, S104, T115, Y142, T145, S156, S158, T159, T163, S169, S170, T176, T178, S184, T189, T193, S201, T202, S204, S205, S206, S219, T226, S230, S232, S233, S234, T240, T249, S252, S253, S255, S266, S268, S272, T279, T292, S305, T308	N284

Putative casein kinase (CKI and CKII) phosphorylation sites are underline and colored in red.

Table S6. Secondary structural components identified in SlHVA22 proteins

No.	Protein name	α- Helix	β- Strand	Coil
1	SlHVA22a	4	-	5
2	SlHVA22b	4	-	5
3	SlHVA22c	4	-	5
4	SlHVA22d	4	-	5
5	SlHVA22e	5	-	6
6	SlHVA22f	13	9	22
7	SlHVA22g	6	-	7
8	SlHVA22h	5	-	6
9	SlHVA22i	4	-	5
10	SlHVA22j	5	-	6
11	SlHVA22k	22	4	27
12	SlHVA22l	4	-	5
13	SlHVA22m	4	-	5
14	SlHVA22n	4	-	5
15	SlHVA22o	5	2	6

Table S7. Secondary structure validation for SlHVA22 proteins through I-TASSER

No.	Locus name	Type	TM-Score	RMSD
1	Solyc01g007780	SlHVA22a	0.37±0.12	12.8±4.2
2	Solyc03g097420	SlHVA22b	0.35±0.12	12.1±4.4
3	Solyc03g110920	SlHVA22c	0.33±0.11	13.4±4.1
4	Solyc03g116350	SlHVA22d	0.48±0.15	9.3±4.6
5	Solyc04g014420	SlHVA22e	0.37±0.13	10.8±4.6
6	Solyc04g076610	SlHVA22f	0.61±0.14	9.0±4.6
7	Solyc04g081340	SlHVA22g	0.28±0.09	16.5±3.0

8	Solyc05g007300	SlHVA22h	0.52±0.15	8.6±4.5
9	Solyc06g072680	SlHVA22i	0.40±0.14	10.5±4.6
10	Solyc10g007820	SlHVA22j	0.42±0.14	10.6±4.6
11	Solyc10g047670	SlHVA22k	0.48±0.15	12.3±4.4
12	Solyc10g051300	SlHVA22l	0.34±0.11	13.2±4.1
13	Solyc10g082040	SlHVA22m	0.25±0.07	16.0±3.2
14	Solyc11g010930	SlHVA22n	0.44±0.14	9.8±4.6
15	Solyc12g089290	SlHVA22o	0.31±0.10	15.5±3.3

TM-score, Template modeling score; RMSD: root-mean-square deviation between residues structurally aligned by TM-align.

Table S8. Parameters for 3D structure modeling of SlHVA22 proteins

No.	Type	C-Score	No. of Decoys	Cluster Density
1	SlHVA22a	- 3.07	922	0.0519
2	SlHVA22b	-3.30	1598	0.0450
3	SlHVA22c	-3.44	1322	0.0413
4	SlHVA22d	-1.92	4470	0.1999
5	SlHVA22e	-3.00	2119	0.0628
6	SlHVA22f	-0.82	600	0.2500
7	SlHVA22g	-4.11	922	0.0173
8	SlHVA22h	-1.63	4406	0.2545
9	SlHVA22i	-2.69	1307	0.0889
10	SlHVA22j	-2.56	1710	0.1036
11	SlHVA22k	-1.95	600	0.0948
12	SlHVA22l	-3.41	1274	0.0411

13	SlHVA22m	-4.41	705	0.0138
14	SlHVA22n	-2.32	1430	0.1254
8	SlHVA22o	-3.74	1157	0.0268

C-Score, confident score.

Table S9. Gene Ontology (GO) annotation for SlHVA22 proteins

Name	Molecular Process	Biological Process	Cellular component
SlHVA22a	protein-transporting ATPase activity, protein binding, cation transmembrane transporter activity	transport	integral component of membrane, plasma membrane
SlHVA22b	protein-transporting ATPase activity, xenobiotic transmembrane transporter activity, antiporter activity, protein binding	establishment of localization, cellular process	cell periphery, intrinsic component of membrane
SlHVA22c	transmembrane transporter activity	cellular process, organic substance transport	integral component of membrane, plasma membrane
SlHVA22d	cytoskeletal protein binding, dystroglycan binding, cell adhesion molecule binding, inorganic cation transmembrane transporter activity	wound healing, cell migration, cell-cell adhesion, exocytosis	intercalated disc, obsolete cell-substrate adherens junction
SlHVA22e	signaling receptor binding, ATP binding,	obsolete multi-organism process, response to stimulus	cytoplasm

	transferase activity		
SlHVA22f	P-type potassium transmembrane transporter activity, sodium ion transmembrane transporter activity, purine ribonucleoside triphosphate binding, ion binding	response to chemical, transition metal ion transport	integral component of membrane
SlHVA22g	ion binding		
SlHVA22h	cell adhesion molecule binding, cytoskeletal protein binding, dystroglycan binding	cell-cell junction assembly, adherens junction organization	obsolete contractile fiber part, adherens junction
SlHVA22i	transition metal ion binding, monooxygenase activity	small molecule metabolic process	
SlHVA22j	transferase activity, metal ion binding	isoprenoid biosynthetic process	
SlHVA22k	ion binding, ion transmembrane transporter activity	response to chemical, transition metal ion transport	plasma membrane, integral component of membrane
SlHVA22l	protein domain specific binding,	aerobic respiration, electron transport	obsolete cytoplasmic

	electron transfer activity	chain	part, intracellular membrane-bounded organelle
SlHVA22l	metal ion binding	metabolic process	Intracellular anatomical structure, membrane
SlHVA22n	guanyl ribonucleotide binding, purine ribonucleoside triphosphate binding	tricarboxylic acid cycle, proton transmembrane transport, G protein-coupled receptor signaling pathway	intrinsic component of membrane
SlHVA22o	signaling receptor binding	anatomical structure formation involved in morphogenesis	plasma membrane

Table S10. Annotated pathways of co-expressed genes with *SlHVA22* genes

Term	MainClass	GeneHitsInSelectedSet	AllGenesInSelectedSet	P-value	GeneListInSelectedSets
Metabolism of terpenoids and polyketides	Metabolism	2	130	0.0085951	[Solyc11g007020, Solyc06g062330]
Oxidative phosphorylation	Metabolism	5	130	0.0094008	[Solyc12g005060, Solyc06g067920, Solyc10g051350, Solyc11g011470, Solyc01g087120]

Pyrimidine metabolism	Metabolism	2	130	0.0141336	[Solyc05g010150, Solyc10g005880]
Metabolism of cofactors and vitamins	Metabolism	3	130	0.0981085	[Solyc12g042460, Solyc07g043590, Solyc12g096590]
Arginine and proline metabolism	Metabolism	1	130	0.1133896	[Solyc07g043590]
Alanine, aspartate and glutamate metabolism	Metabolism	2	130	0.1635698	[Solyc10g080320, Solyc10g078550]
Nucleotide metabolism	Metabolism	4	130	0.1932533	[Solyc10g080320, Solyc08g079020, Solyc05g010150, Solyc10g005880]
Carbohydrate metabolism	Metabolism	6	130	0.2612796	[Solyc06g071920, Solyc07g007790, Solyc12g049590, Solyc03g097410, Solyc11g008720, Solyc09g090140]
Glyoxylate and dicarboxylate metabolism	Metabolism	1	130	0.5853759	[Solyc09g090140]
Nitrogen metabolism	Metabolism	2	130	0.6316198	[Solyc10g078550, Solyc06g074990]
Phenylalanine metabolism	Metabolism	1	130	0.6316198	[Solyc08g068680]
Energy metabolism	Metabolism	12	130	0.7178608	[Solyc06g071920, Solyc12g005060, Solyc06g067920, Solyc02g011990, Solyc10g051350, Solyc08g067320, Solyc11g011470, Solyc10g078550, Solyc01g087120, Solyc06g074990, Solyc09g064580, Solyc09g090140]
Purine metabolism	Metabolism	3	130	0.7814045	[Solyc10g080320, Solyc08g079020, Solyc05g010150]
Pantothenate and Cobiosynthesis	Metabolism	1	130	0.9898759	[Solyc07g043590]
Stilbenoid, diarylheptanoid and gingerol biosynthesis	Metabolism	1	130	0.011852	[Solyc12g088170]

Carbon fixation in photosynthetic organisms	Metaboli sm	2	130	0.015 8787	[Solyc06g071920, Solyc09g090140]
Citrate cycle (TCcycle)	Metaboli sm	1	130	0.031 2843	[Solyc09g090140]
Starch and sucrose metabolism	Metaboli sm	2	130	0.041 388	[Solyc07g007790, Solyc11g008720]
Biosynthesis of other secondary metabolites	Metaboli sm	3	130	0.053 2613	[Solyc12g088170, Solyc12g042460, Solyc11g008720]
Glycan biosynthesis and metabolism	Metaboli sm	1	130	0.161 8181	[Solyc08g076570]
Photosynthesis	Metaboli sm	3	130	0.198 2193	[Solyc12g005060, Solyc02g011990, Solyc09g064580]
Glycerolipid metabolism	Metaboli sm	1	130	0.357 6286	[Solyc09g014350]
Selenocompound metabolism	Metaboli sm	1	130	0.719 5953	[Solyc10g081510]
Cysteine and methionine metabolism	Metaboli sm	2	130	2.92E -03	[Solyc10g081510, Solyc09g090140]
Glycerophospholipid metabolism	Metaboli sm	2	130	0.017 7595	[Solyc09g014350, Solyc07g055980]
Terpenoid backbone biosynthesis	Metaboli sm	1	130	0.030 3528	[Solyc11g007020]
Metabolism of other amino acids	Metaboli sm	4	130	0.077 0684	[Solyc09g065900, Solyc10g081510, Solyc07g043590, Solyc11g008720]
Inositol phosphate metabolism	Metaboli sm	2	130	0.271 6742	[Solyc12g049590, Solyc03g097410]
Pyruvate metabolism	Metaboli sm	1	130	0.274 5623	[Solyc09g090140]

Glycolysis / Gluconeogenesis	Metabolism	1	130	0.396317	[Solyc06g071920]
Metabolism	Metabolism	34	130	0.4650306	[Solyc11g007020, Solyc12g005060, Solyc10g080320, Solyc01g079390, Solyc10g081510, Solyc05g010150, Solyc10g005880, Solyc12g096990, Solyc07g055980, Solyc12g096590, Solyc06g062330, Solyc09g065900, Solyc12g042460, Solyc06g067920, Solyc08g067320, Solyc07g007790, Solyc12g049590, Solyc07g043590, Solyc02g011990, Solyc09g014350, Solyc08g079020, Solyc10g078550, Solyc06g074990, Solyc03g097410, Solyc08g076570, Solyc11g008720, Solyc09g090140, Solyc12g088170, Solyc06g071920, Solyc10g051350, Solyc11g011470, Solyc08g068680, Solyc01g087120, Solyc09g064580]
beta-Alanine metabolism	Metabolism	1	130	0.512635	[Solyc07g043590]
Riboflavin metabolism	Metabolism	1	130	0.5862166	[Solyc12g096590]
Ubiquinone and other terpenoid-quinone biosynthesis	Metabolism	1	130	0.6155432	[Solyc12g042460]
Zeatin biosynthesis	Metabolism	1	130	0.6257011	[Solyc06g062330]
Cyanoamino acid metabolism	Metabolism	1	130	0.8026207	[Solyc11g008720]
Flavonoid biosynthesis	Metabolism	1	130	0.8102446	[Solyc12g088170]
Lipid metabolism	Metabolism	2	130	8.85E-05	[Solyc09g014350, Solyc07g055980]

Arginine biosynthesis	Metabolism	1	130	0.0030303	[Solyc10g078550]
Lysine degradation	Metabolism	2	130	0.1494335	[Solyc01g079390, Solyc12g096990]
Photosynthesis - antennproteins	Metabolism	1	130	0.2157282	[Solyc08g067320]
Amino acid metabolism	Metabolism	8	130	0.2940862	[Solyc10g080320, Solyc01g079390, Solyc10g081510, Solyc12g096990, Solyc10g078550, Solyc08g068680, Solyc07g043590, Solyc09g090140]
Glycosylphosphatidylinositol (GPI)-anchor biosynthesis	Metabolism	1	130	0.5276394	[Solyc08g076570]
Glutathione metabolism	Metabolism	1	130	0.6706342	[Solyc09g065900]
Phenylpropanoid biosynthesis	Metabolism	3	130	0.2718919	[Solyc12g088170, Solyc12g042460, Solyc11g008720]
Ribosome biogenesis in eukaryotes	Genetic Information Processing	1	130	0.0395269	[Solyc07g063270]
Folding, sorting and degradation	Genetic Information Processing	14	130	0.0580914	[Solyc02g082460, Solyc10g012240, Solyc06g005990, Solyc06g082600, Solyc05g056110, Solyc10g077030, Solyc02g078210, Solyc08g008130, Solyc09g082320, Solyc11g045130, Solyc07g016200, Solyc06g084210, Solyc11g019920, Solyc10g084050]
Translation	Genetic Information Processing	11	130	0.0771657	[Solyc09g009120, Solyc09g030390, Solyc09g075290, Solyc12g096540, Solyc12g008570, Solyc12g035650, Solyc09g092090, Solyc07g006010, Solyc12g014600, Solyc07g063270, Solyc10g018610]

Ubiquitin mediated proteolysis	Genetic Information Processing	4	130	0.1757509	[Solyc08g008130, Solyc10g012240, Solyc06g082600, Solyc02g078210]
Protein processing in endoplasmic reticulum	Genetic Information Processing	4	130	0.2853175	[Solyc06g082600, Solyc06g084210, Solyc11g019920, Solyc10g084050]
RNdegradation	Genetic Information Processing	4	130	0.3562572	[Solyc02g082460, Solyc11g045130, Solyc06g005990, Solyc05g056110]
mRNsurveillance pathway	Genetic Information Processing	3	130	0.4104152	[Solyc09g009120, Solyc09g030390, Solyc12g014600]
Ribosome	Genetic Information Processing	4	130	0.5026504	[Solyc09g075290, Solyc12g096540, Solyc09g092090, Solyc10g018610]
Base excision repair	Genetic Information Processing	1	130	0.5335866	[Solyc06g076660]

Nucleotide excision repair	Genetic Information Processing	2	130	0.67289	[Solyc06g076660, Solyc02g067670]
Basal transcription factors	Genetic Information Processing	1	130	0.9663864	[Solyc10g079370]
Genetic Information Processing	Genetic Information Processing	33	130	0.0014094	[Solyc02g082460, Solyc06g076660, Solyc12g008570, Solyc12g035650, Solyc09g092090, Solyc06g082600, Solyc05g056110, Solyc10g077030, Solyc10g079370, Solyc09g082320, Solyc11g045130, Solyc11g044340, Solyc07g008880, Solyc10g018610, Solyc11g019920, Solyc01g067830, Solyc10g012240, Solyc06g005990, Solyc02g078210, Solyc09g009120, Solyc09g030390, Solyc08g008130, Solyc09g009300, Solyc09g075290, Solyc12g096540, Solyc07g016200, Solyc06g084210, Solyc07g006010, Solyc03g033600, Solyc12g014600, Solyc07g063270, Solyc02g067670, Solyc10g084050]
Replication and repair	Genetic Information Processing	2	130	0.0073591	[Solyc06g076660, Solyc02g067670]
Mismatch repair	Genetic Information Processing	1	130	0.3563425	[Solyc06g076660]

Aminoacyl-tRNbiosynthesis	Genetic Information Processing	1	130	0.4682645	[Solyc12g008570]
Transcription	Genetic Information Processing	6	130	0.9521377	[Solyc01g067830, Solyc09g009300, Solyc11g044340, Solyc07g008880, Solyc03g033600, Solyc10g079370]
DNreplication	Genetic Information Processing	1	130	1.41E-07	[Solyc06g076660]
Nucleocytoplasmic transport	Genetic Information Processing	3	130	0.7579823	[Solyc09g030390, Solyc12g035650, Solyc07g006010]
Spliceosome	Genetic Information Processing	5	130	0.0082091	[Solyc01g067830, Solyc09g009300, Solyc11g044340, Solyc07g008880, Solyc03g033600]
Proteasome	Genetic Information Processing	3	130	0.3625192	[Solyc09g082320, Solyc07g016200, Solyc10g077030]

Environmental Information Processing	Environ mental Informati on Processin g	8	130	0.005 4095	[Solyc09g008240, Solyc02g090390, Solyc12g099830, Solyc07g064820, Solyc08g081610, Solyc12g049590, Solyc03g097410, Solyc02g071350]
Membrane transport	Environ mental Informati on Processin g	2	130	0.023 7072	[Solyc09g008240, Solyc02g071350]
Phosphatidylinositol signaling system	Environ mental Informati on Processin g	2	130	0.131 8688	[Solyc12g049590, Solyc03g097410]
Plant hormone signal transduction	Environ mental Informati on Processin g	2	130	0.615 7035	[Solyc02g090390, Solyc12g099830]
MAPK signaling pathway - plant	Environ mental Informati on Processin g	3	130	0.344 657	[Solyc02g090390, Solyc07g064820, Solyc08g081610]
ABC transporters	Environ mental	2	130	1.17E -02	[Solyc09g008240, Solyc02g071350]

	Information Processing				
Signal transduction	Environmental Information Processing	6	130	0.3902295	[Solyc02g090390, Solyc12g099830, Solyc07g064820, Solyc08g081610, Solyc12g049590, Solyc03g097410]
Cellular Processes	Cellular Processes	3	130	0.2123418	[Solyc12g099080, Solyc03g031650, Solyc12g049590]
Endocytosis	Cellular Processes	1	130	0.0716251	[Solyc12g099080]
Phagosome	Cellular Processes	1	130	0.5704954	[Solyc12g049590]
Transport and catabolism	Cellular Processes	3	130	1.72E-04	[Solyc12g099080, Solyc03g031650, Solyc12g049590]
Autophagy - other	Cellular Processes	1	130	0.3710518	[Solyc03g031650]
Plant-pathogen interaction	Organismal Systems	2	130	0.990845	[Solyc08g081610, Solyc07g047960]
Organismal Systems	Organismal Systems	2	130	0.8102446	[Solyc08g081610, Solyc07g047960]
Environmental adaptation	Organismal Systems	2	130	0.4452339	[Solyc08g081610, Solyc07g047960]

Protein kinases	Brite Hierarchies	4	130	0.005674	[Solyc02g090390, Solyc12g099830, Solyc07g064820, Solyc06g073630]
Ion channels	Brite Hierarchies	2	130	0.0605392	[Solyc07g052390, Solyc07g007690]
Peptidases and inhibitors	Brite Hierarchies	5	130	0.1103079	[Solyc10g024320, Solyc09g082320, Solyc07g016200, Solyc10g077030, Solyc02g079000]
Transcription machinery	Brite Hierarchies	5	130	0.1175514	[Solyc09g009120, Solyc02g094360, Solyc06g083260, Solyc11g012740, Solyc10g079370]
DNreplication proteins	Brite Hierarchies	4	130	0.1780005	[Solyc06g076660, Solyc10g049360, Solyc03g033600, Solyc07g066660]
Messenger RNbiogenesis	Brite Hierarchies	14	130	0.2174993	[Solyc02g082460, Solyc05g032850, Solyc12g035650, Solyc04g077060, Solyc06g005990, Solyc01g090480, Solyc05g056110, Solyc12g040510, Solyc09g009120, Solyc09g030390, Solyc11g045130, Solyc07g006010, Solyc12g014600, Solyc10g084050]
GTP-binding proteins	Brite Hierarchies	1	130	0.2693477	[Solyc12g099080]
Glycosyltransferases	Brite Hierarchies	1	130	0.3598012	[Solyc01g073750]
Cytoskeleton proteins	Brite Hierarchies	2	130	0.5040355	[Solyc08g008130, Solyc12g098630]

Lipid biosynthesis proteins	Brite Hierarchies	1	130	0.6367832	[Solyc09g014350]
Ribosome	Brite Hierarchies	4	130	0.7257998	[Solyc09g075290, Solyc12g096540, Solyc09g092090, Solyc10g018610]
Amino acid related enzymes	Brite Hierarchies	1	130	0.7447201	[Solyc12g008570]
Translation factors	Brite Hierarchies	2	130	0.9276829	[Solyc07g008610, Solyc11g045120]
Protein families: signaling and cellular processes	Brite Hierarchies	22	130	0.011852	[Solyc12g099080, Solyc12g005270, Solyc07g052390, Solyc10g079200, Solyc08g079020, Solyc10g078550, Solyc06g074990, Solyc02g071350, Solyc12g040510, Solyc09g008240, Solyc08g081190, Solyc06g071920, Solyc08g008130, Solyc02g070530, Solyc12g098630, Solyc05g009690, Solyc07g007690, Solyc04g081150, Solyc09g008490, Solyc10g054930, Solyc11g019920, Solyc10g084050]
Transcription factors	Brite Hierarchies	5	130	0.0312843	[Solyc09g009100, Solyc08g081610, Solyc10g054330, Solyc07g047960, Solyc01g073910]
Protein phosphatases and associated proteins	Brite Hierarchies	4	130	0.0394022	[Solyc04g074190, Solyc04g081900, Solyc07g007690, Solyc04g056340]
Protein families: metabolism	Brite Hierarchies	20	130	0.117494	[Solyc04g074190, Solyc12g008570, Solyc12g005060, Solyc02g011990, Solyc06g073630, Solyc04g081900, Solyc09g014350, Solyc10g077030, Solyc02g079000, Solyc04g056340, Solyc02g090390, Solyc01g073750,

					Solyc10g024320, Solyc09g082320, Solyc12g099830, Solyc07g064820, Solyc07g016200, Solyc08g067320, Solyc07g007690, Solyc09g064580]
Domain-containing proteins not elsewhere classified	Brite Hierarchies	1	130	0.1417706	[Solyc09g008490]
Photosynthesis proteins	Brite Hierarchies	4	130	0.1485029	[Solyc12g005060, Solyc02g011990, Solyc08g067320, Solyc09g064580]
Transporters	Brite Hierarchies	8	130	0.1512258	[Solyc09g008240, Solyc08g081190, Solyc02g070530, Solyc10g079200, Solyc05g009690, Solyc06g074990, Solyc10g054930, Solyc02g071350]
Chromosome and associated proteins	Brite Hierarchies	9	130	0.1987293	[Solyc11g066160, Solyc05g021530, Solyc12g005270, Solyc01g079390, Solyc07g006010, Solyc10g049360, Solyc12g096990, Solyc04g081150, Solyc07g066660]
Exosome	Brite Hierarchies	11	130	0.2874254	[Solyc09g008240, Solyc12g099080, Solyc06g071920, Solyc12g005270, Solyc08g079020, Solyc10g078550, Solyc04g081150, Solyc02g071350, Solyc12g040510, Solyc11g019920, Solyc10g084050]
Brite Hierarchies	Brite Hierarchies	105	130	0.5862166	[Solyc06g076660, Solyc07g008610, Solyc08g081610, Solyc02g094360, Solyc12g014470, Solyc12g098630, Solyc11g045130, Solyc10g054930, Solyc11g019920, Solyc05g032850, Solyc04g074190, Solyc05g054100, Solyc04g077060, Solyc06g005990, Solyc02g011990, Solyc06g074990, Solyc02g079000, Solyc12g009610, Solyc12g040510, Solyc09g030390, Solyc06g071920, Solyc02g090390, Solyc10g024320, Solyc09g009300, Solyc07g064820, Solyc05g021530, Solyc02g067670, Solyc10g084050, Solyc04g008510, Solyc08g023590, Solyc06g082600, Solyc07g052390, Solyc12g096990, Solyc10g077030, Solyc07g008880, Solyc12g049590, Solyc04g081150, Solyc07g007810, Solyc01g067830,

					Solyc12g005270, Solyc10g079200, Solyc02g078210, Solyc09g009120, Solyc11g066160, Solyc08g008130, Solyc09g075290, Solyc11g045120, Solyc11g019930, Solyc06g084210, Solyc03g083020, Solyc07g066660, Solyc07g063270, Solyc11g012740, Solyc02g082460, Solyc12g099080, Solyc12g035650, Solyc10g054330, Solyc06g073630, Solyc10g076360, Solyc01g090480, Solyc11g068450, Solyc01g073750, Solyc01g073910, Solyc07g007690, Solyc05g026130, Solyc10g083600, Solyc04g056340, Solyc12g096540, Solyc09g009100, Solyc10g049360, Solyc12g014600, Solyc06g083260, Solyc09g008490, Solyc09g064580, Solyc12g008570, Solyc09g092090, Solyc12g005060, Solyc01g079390, Solyc03g031650, Solyc05g056110, Solyc02g071350, Solyc10g079370, Solyc09g008240, Solyc08g081190, Solyc09g082320, Solyc11g044340, Solyc08g067320, Solyc05g009690, Solyc10g018610, Solyc10g012240, Solyc04g081900, Solyc09g014350, Solyc08g079020, Solyc10g078550, Solyc08g062560, Solyc02g070530, Solyc12g099830, Solyc09g091830, Solyc12g044370, Solyc09g090740, Solyc01g090190, Solyc07g016200, Solyc07g047960, Solyc07g006010, Solyc03g033600]
Mitochondrial biogenesis	Brite Hierarchies	4	130	0.6059362	[Solyc10g024320, Solyc12g008570, Solyc12g014470, Solyc03g031650]
Protein families: genetic information processing	Brite Hierarchies	79	130	6.34E-05	[Solyc02g082460, Solyc06g076660, Solyc12g099080, Solyc07g008610, Solyc12g035650, Solyc08g081610, Solyc10g054330, Solyc10g076360, Solyc01g090480, Solyc02g094360, Solyc11g068450, Solyc12g014470, Solyc11g045130, Solyc01g073910, Solyc11g019920, Solyc05g032850, Solyc05g026130, Solyc05g054100, Solyc04g077060, Solyc06g005990, Solyc10g083600, Solyc02g079000, Solyc12g009610, Solyc12g040510,

					Solyc09g030390, Solyc06g071920, Solyc10g024320, Solyc09g009300, Solyc12g096540, Solyc05g021530, Solyc09g009100, Solyc10g049360, Solyc12g014600, Solyc06g083260, Solyc02g067670, Solyc10g084050, Solyc04g008510, Solyc08g023590, Solyc12g008570, Solyc09g092090, Solyc06g082600, Solyc01g079390, Solyc03g031650, Solyc12g096990, Solyc05g056110, Solyc10g077030, Solyc10g079370, Solyc09g082320, Solyc11g044340, Solyc07g008880, Solyc05g009690, Solyc12g049590, Solyc04g081150, Solyc10g018610, Solyc07g007810, Solyc01g067830, Solyc10g012240, Solyc12g005270, Solyc02g078210, Solyc09g009120, Solyc11g066160, Solyc08g062560, Solyc08g008130, Solyc09g075290, Solyc09g091830, Solyc12g044370, Solyc09g090740, Solyc01g090190, Solyc07g016200, Solyc11g045120, Solyc11g019930, Solyc06g084210, Solyc07g047960, Solyc07g006010, Solyc03g033600, Solyc03g083020, Solyc07g066660, Solyc07g063270, Solyc11g012740]
CD molecules	Brite Hierarchies	2	130	0.3393994	[Solyc09g008240, Solyc02g071350]
Transfer RNbiogenesis	Brite Hierarchies	3	130	0.3951042	[Solyc12g008570, Solyc12g044370, Solyc03g083020]
Proteasome	Brite Hierarchies	3	130	0.512635	[Solyc09g082320, Solyc07g016200, Solyc10g077030]
Membrane trafficking	Brite Hierarchies	14	130	0.5977576	[Solyc12g099080, Solyc07g007810, Solyc05g026130, Solyc05g054100, Solyc03g031650, Solyc10g076360, Solyc12g009610, Solyc06g071920, Solyc11g068450,

					Solyc06g084210, Solyc05g009690, Solyc12g049590, Solyc11g019920, Solyc10g084050]
Spliceosome	Brite Hierarchies	7	130	0.0855238	[Solyc09g030390, Solyc01g067830, Solyc09g009300, Solyc01g090190, Solyc11g044340, Solyc07g008880, Solyc03g033600]
Ribosome biogenesis	Brite Hierarchies	5	130	0.2052012	[Solyc08g062560, Solyc09g090740, Solyc10g083600, Solyc07g063270, Solyc10g084050]
Ubiquitin system	Brite Hierarchies	7	130	0.191198	[Solyc04g008510, Solyc08g008130, Solyc10g012240, Solyc06g082600, Solyc03g031650, Solyc11g019930, Solyc02g078210]
DNrepair and recombination proteins	Brite Hierarchies	3	130	0.2461736	[Solyc06g076660, Solyc08g023590, Solyc02g067670]
Chaperones and folding catalysts	Brite Hierarchies	4	130	0.3373161	[Solyc09g091830, Solyc12g014470, Solyc02g079000, Solyc11g019920]

Table S11. In silico subcellular localization of SlHVA22 proteins

SlHVA22a WoLFPSORT prediction nucl: 9, cyto: 2, extr: 1, cysk: 1, golg: 1

PSORT features and traditional PSORTII prediction

No.14 Nearest Neighbors

id site distance identity comments

1	At2g39250.1	nucl	177.162	12.95%	[Arath]
2	WR29_ARATH	nucl	194.499	13.49%	[Uniprot] SWISS-PROT45:Nuclear.
3	At1g33030.1	cyto	196.351	14.20%	[Arath]
4	At1g22190.1	nucl	208.715	14.18%	[Arath]
5	ILLA_LEUGL	extr	217.269	11.36%	[Uniprot] SWISS-PROT45:Secreted.
6	At4g39780.1	nucl	223.392	12.13%	[Arath]
7	At5g65130.1	nucl	227.6	13.00%	[Arath]
8	At2g43130.1	golg	247.16	9.91%	[Arath]
9	At1g18450.1	nucl	249.344	14.29%	[Arath]
10	At1g64380.1	nucl	252.787	13.13%	[Arath]
11	RPB1_ARATH	nucl	256.002	4.84%	[Uniprot] SWISS-PROT45:Nuclear.
12	TTG1_ARATH	cyto	258.983	15.20%	[Uniprot] SWISS-PROT45:Cytoplasmic.
13	At3g60490.1	nucl	263.043	12.84%	[Arath]
14	At5g62500.1	cysk	264.822	11.60%	[Arath]

SlHVA22b WoLFPSORT prediction extr: 6, vacu: 3, nucl: 1, cyto: 1, mito: 1, plas: 1, golg: 1

PSORT features and traditional PSORTII prediction

14 Nearest Neighbors

No.	id	site	distance	identity	comments
1	E13A_LYCES	extr	215.85	11.61%	[Uniprot] SWISS-PROT45:Extracellular.
2	At2g29960.1	cyto	251.258	12.44%	[Arath]
3	AFP4_RAPSA	extr	331.72	11.03%	[Uniprot] SWISS-PROT45:Secreted.
4	PHS1_PHALU	vacu	346.209	9.11%	[Uniprot] SWISS-PROT45:Cotyledonary membrane-bound vacuolar protein bodies.
5	GAS1_ARATH	extr	349.568	13.24%	[Uniprot] SWISS-PROT45:Secreted.
6	At1g21230.1	plas	357.998	6.41%	[Arath]
7	At5g17020.1	nucl	367.876	5.77%	[Arath]
8	VCLC_PEA	vacu	381.164	8.93%	[Uniprot] SWISS-PROT45:Cotyledonary membrane-bound vacuolar protein bodies.
9	AFP3_BRANA	extr	388.604	10.29%	[Uniprot] SWISS-PROT45:Secreted.
10	STSY_CATRO	vacu	394.169	11.08%	[Uniprot] SWISS-PROT45:Vacuolar.
11	ADT1_WHEAT	mito	402.523	9.97%	[Uniprot] SWISS-PROT45:Integral membrane protein. Mitochondrial inner membrane.
12	EXTN_TOBAC	extr	405.401	5.97%	[Uniprot] SWISS-PROT45:Extracellular matrix.
13	AFP1_BRANA	extr	405.71	12.50%	[Uniprot] SWISS-PROT45:Secreted.
14	At4g38240.1	golg	406.638	11.26%	[Arath]

SlHVA22c WoLFPSORT prediction E.R.: 4.5, E.R._plas: 4, nucl: 3, plas: 2.5, vacu: 2, chlo: 1, pero: 1

PSORT features and traditional PSORTII prediction

14 Nearest Neighbors

No.	id	site	distance	identity	comments
1	At2g23800.1	E.R.	240.68	13.56%	[Arath]
2	SPR1_IPOBA	vacu	269.291	12.61%	[Uniprot] SWISS-PROT45:Vacuolar.
3	At2g39970.1	pero	289.15	11.48%	[Arath]
4	At5g17020.1	nucl	323.826	6.42%	[Arath]
5	NCPR_PHAAU	E.R.	329.298	8.99%	[Uniprot] SWISS-PROT45:Endoplasmic reticulum. Anchored to the ER membrane by its N-terminal hydrophobic region.
6	At5g53470.1	plas	344.03	14.50%	[Arath]
7	At4g27780.1	E.R._plas	350.213	13.56%	[Arath]
8	At1g21270.1	plas	357.245	9.56%	[Arath]
9	SPRA_IPOBA	vacu	357.791	12.33%	[Uniprot] SWISS-PROT45:Vacuolar.
10	MAOC_FLATR	chlo	380.503	10.34%	[Uniprot] SWISS-PROT45:Chloroplast.
11	At2g18640.1	E.R.	381.884	13.17%	[Arath]
12	SC11_ARATH	nucl	383.116	8.61%	[Uniprot] SWISS-PROT45:Nuclear.
13	NTPA_PEA	nucl	387.156	10.11%	[Uniprot] SWISS-PROT45:Nuclear.

14 At4g24520.1 E.R. 389.521 8.95% [Arath]

SlHVA22d WoLFPSORT prediction extr: 4, vacu: 4, E.R.: 2, chlo: 1, cyto: 1, mito: 1, pero: 1

PSORT features and traditional PSORTII prediction

14 Nearest Neighbors

No.	id	site	distance	identity	comments
1	At2g29960.1	cyto	387.426	12.44%	[Arath]
2	MFPA_CUCSA	pero	402.102	9.24%	[Uniprot] SWISS-PROT45:Glyoxysomal. GO:0009514; C:glyoxysome; Evidence:NAS
3	At1g20330.1	E.R.	434.216	14.68%	[Arath]
4	At3g10570.1	mito	438.147	9.16%	[Arath]
5	CKX3_ARATH	extr	438.25	11.28%	[Uniprot] SWISS-PROT45:Extracellular.
6	At1g77590.1	chlo	459.169	8.25%	[Arath]
7	BG1_ARATH	E.R.	464.551	10.80%	[Uniprot] SWISS-PROT45:Endoplasmic reticulum.
8	ASPR_CUCPE	vacu	469.784	10.53%	[Uniprot] SWISS-PROT45:Vacuolar.
9	E13H_TOBAC	extr	471.476	13.57%	[Uniprot] SWISS-PROT45:Extracellular.
10	E13A_LYCES	extr	472.463	12.20%	[Uniprot] SWISS-PROT45:Extracellular.

11 VPEG_ARATH vacu 474.38 9.80% [Uniprot] SWISS-PROT45:Vacuolar.

12 AFP4_RAPSA extr 478.689 13.64% [Uniprot] SWISS-PROT45:Secreted.

13 At4g35350.1 vacu 480.851 13.24% [Arath]

14 VPEB_ARATH vacu 481.14 10.70% [Uniprot] SWISS-PROT45:Vacuolar.

SlHVA22e WoLFPSORT prediction cyto_nucl: 6.33333, cyto: 5, nucl: 3.5, chlo: 3, cysk_nucl: 2.83333, mito: 2

PSORT features and traditional PSORTII prediction

14 Nearest Neighbors

No.id site distance identity comments

1 UBIQ_ACECL cyto_nucl 250.703 14.55% [Uniprot] SWISS-PROT45:Nuclear and cytoplasmic.

2 UBIQ_ARATH cyto_nucl 275.769 12.73% [Uniprot] SWISS-PROT45:Nuclear and cytoplasmic.

3 UBIQ_CHLRE cyto_nucl 275.769 13.64% [Uniprot] SWISS-PROT45:Nuclear and cytoplasmic.

4 UBIQ_SOYBN cyto_nucl 275.769 12.73% [Uniprot] SWISS-PROT45:Nuclear and cytoplasmic.

5 At3g51260.1 cysk_nucl 291.017 16% [Arath]

6 At1g04640.1 mito 294.507 10.64% [Arath]

7 RPBX_BRANA nucl 294.525 13.64% [Uniprot] SWISS-PROT45:Nuclear.

8 GSHR_PEA cyto 305.931 7.03% [Uniprot] SWISS-PROT45:Cytoplasmic.

9 KADC_MAIZE chlo 312.751 14.41% [Uniprot] SWISS-PROT45:Chloroplast.

10 At4g34050.1 cyto 313.426 12.36% [Arath]

11 At4g29430.1 mito 314.941 16.28% [Arath]

12 At1g67990.1 cyto 316.814 15.02% [Arath]

13 CRTI_SOYBN chlo 319.005 6.49% [Uniprot] SWISS-PROT45:Chloroplast; chromoplast.

14 RBMT_PEA chlo 319.104 8.18% [Uniprot] SWISS-PROT45:Chloroplast.

SlHVA22f WoLFPSORT prediction nucl: 12, extr: 2

PSORT features and traditional PSORTII prediction

14 Nearest Neighbors

No.id site distance identity comments

1 At4g05420.1 nucl 418.573 11.49% [Arath]

2 PGL2_JUNAS extr 645.461 13.81% [Uniprot] SWISS-PROT45:Secreted.

3 PGLR_ACTCH extr 744.1 9.24% [Uniprot] SWISS-PROT45:Secreted.

4 TBP2_ARATH nucl 745.091 10.23% [Uniprot] SWISS-PROT45:Nuclear.

5 TBP1_ARATH nucl 760.569 10.23% [Uniprot] SWISS-PROT45:Nuclear.

6 TBP2_MAIZE nucl 777.428 10.23% [Uniprot] SWISS-PROT45:Nuclear.

7 TBP_SOYBN nucl 782.333 10.02% [Uniprot] SWISS-PROT45:Nuclear.

8	TBP_TOBAC	nucl	792.556	10.45%	[Uniprot] SWISS-PROT45:Nuclear.
9	TBP1_MAIZE	nucl	801.295	10.02%	[Uniprot] SWISS-PROT45:Nuclear.
10	TBP1_WHEAT	nucl	802.263	11.73%	[Uniprot] SWISS-PROT45:Nuclear.
11	TBP_MESCR	nucl	802.758	9.59%	[Uniprot] SWISS-PROT45:Nuclear.
12	TBP_SOLTU	nucl	809.91	10.02%	[Uniprot] SWISS-PROT45:Nuclear.
13	TBP2_WHEAT	nucl	867.618	10.66%	[Uniprot] SWISS-PROT45:Nuclear.
14	TBP_ACECL	nucl	896.678	11.73%	[Uniprot] SWISS-PROT45:Nuclear.

SlHVA22g WoLFPSORT prediction chlo: 9, nucl: 4, cyto: 1

PSORT features and traditional PSORTII prediction

14 Nearest Neighbors

No.	id	site	distance	identity	comments
1	At1g06680.1	chlo	214.159	12.79%	[Arath] Subclass:thylakoid
2	RBS5_ACECL	chlo	234.773	12.17%	[Uniprot] SWISS-PROT45:Chloroplast.
3	At1g75080.1	nucl	255.792	11.01%	[Arath]
4	TRB2_ARATH	cyto	265.066	12.53%	[Uniprot] SWISS-PROT45:Cytoplasmic.
5	RBS2_CHLRE	chlo	268.684	11.18%	[Uniprot] SWISS-PROT45:Chloroplast.
6	FER2_SPIOL	chlo	271.163	8.88%	[Uniprot] SWISS-PROT45:Chloroplast.

7 RBS_LARLA chlo 277.188 11.18% [Uniprot] SWISS-PROT45:Chloroplast.

8 At3g14230.1 nucl 282.523 14.78% [Arath]

9 RBS5_ACEME chlo 283.165 11.18% [Uniprot] SWISS-PROT45:Chloroplast.

10 At1g53910.1 nucl 284.295 13.69% [Arath]

11 ALFC_CHLRE chlo 285.83 12.57% [Uniprot] SWISS-PROT45:Chloroplast.

12 RBS3_ACEME chlo 286.93 12.50% [Uniprot] SWISS-PROT45:Chloroplast.

13 At5g50080.1 nucl 287.647 13.82% [Arath]

14 ALFC_ORYSA chlo 288.087 14.32% [Uniprot] SWISS-PROT45:Chloroplast.

SlHVA22h WoLFPSORT prediction E.R.: 3.5, cyto: 3, E.R._plas: 3, mito: 2, plas: 1.5, nucl: 1, extr: 1, vacu: 1, pero: 1

PSORT features and traditional PSORTII prediction

14 Nearest Neighbors

No.id site distance identity comments

1 ADT_CHLRE mito 230.755 11.36% [Uniprot] SWISS-PROT45:Integral membrane protein. Mitochondrial inner membrane.

2 At5g53470.1 plas 270.988 12.13% [Arath]

3 At4g27780.1 E.R._plas 327.684 12.99% [Arath]

4 MAOX_MESCR cyto 355.97 9.23% [Uniprot] SWISS-PROT45:Cytoplasmic.

5 At2g23800.1 E.R. 366.678 11.17% [Arath]

6 MFPA_CUCSA pero 384.506 9.10% [Uniprot] SWISS-PROT45:Glyoxysomal. GO:0009514; C:glyoxysome; Evidence:NAS

7 At1g20330.1 E.R. 384.68 13.85% [Arath]

8 F16Q_BRANA cyto 391.039 13.27% [Uniprot] SWISS-PROT45:Cytoplasmic.

9 E13A_LYCES extr 391.103 13.99% [Uniprot] SWISS-PROT45:Extracellular.

10 At3g30775.1 mito 393.43 10.37% [Arath]

11 NCPR_CATRO E.R. 395.882 8.96% [Uniprot] SWISS-PROT45:Endoplasmic reticulum. Anchored to the ER membrane by its N-terminal hydrophobic region.

12 At5g17020.1 nucl 398.479 5.40% [Arath]

13 F16Q_SPIOL cyto 398.479 14.08% [Uniprot] SWISS-PROT45:Cytoplasmic.

14 TIP2_TOBAC vacu 406.185 12.40% [Uniprot] SWISS-PROT45:Integral membrane protein; tonoplast membrane.

SlHVA22i WoLFPSORT prediction chlo: 11, cyto: 1.5, cyto_E.R.: 1.5, nucl_plas: 1

PSORT features and traditional PSORTII prediction

14 Nearest Neighbors

No.id site distance identity comments

1 CB2_PHYPA chlo 225.936 12.27% [Uniprot] SWISS-PROT45:Chloroplast thylakoid membrane.

2 CB23_POLMU chlo 240.94 14.72% [Uniprot] SWISS-PROT45:Chloroplast thylakoid membrane.

3 At1g56330.1 cyto_E.R. 252.278 16.06% [Arath]

4 PSY_CUCME chlo 259.901 10.19% [Uniprot] SWISS-PROT45:Chloroplast.

5 CRTI_ARATH chlo 261.119 9.72% [Arath] [Uniprot] SWISS-PROT45:Chloroplast; chromoplast. Evidence:TAS Pubmed:9700076

6 AAT5_ARATH chlo 267.813 10.82% [Arath] [Uniprot] SWISS-PROT45:Chloroplast. Evidence:IDA Pubmed:7766905

7 CB22_HORVU chlo 269.008 14.02% [Uniprot] SWISS-PROT45:Chloroplast thylakoid membrane.

8 CB28_PEA chlo 269.629 13.81% [Uniprot] SWISS-PROT45:Chloroplast thylakoid membrane.

9 PODK_FLATR chlo 275.895 5.14% [Uniprot] SWISS-PROT45:Chloroplast.

10 CB2A_SPIOL chlo 276.431 11.61% [Uniprot] SWISS-PROT45:Chloroplast thylakoid membrane.

11 At4g25100.1 chlo 277.09 12.26% [Arath]

12 SODC_PANGI cyto 279.73 9.21% [Uniprot] SWISS-PROT45:Cytoplasmic.

13 PODK_FLABI chlo 279.852 4.93% [Uniprot] SWISS-PROT45:Chloroplast.

14 At5g65430.1 nucl_plas 287.866 14.11% [Arath]

SlHVA22j WoLFPSORT prediction chlo: 8, extr: 2, vacu: 2, nucl: 1, plas: 1

PSORT features and traditional PSORTII prediction

14 Nearest Neighbors

No.	id	site	distance	identity	comments
1	KAP1_ARATH	chlo	355.038	14.13%	[Uniprot] SWISS-PROT45:Chloroplast.
2	CB11_LYCES	chlo	355.363	12.60%	[Uniprot] SWISS-PROT45:Chloroplast thylakoid membrane.
3	GAS3_ARATH	extr	363.373	13.46%	[Uniprot] SWISS-PROT45:Secreted.
4	CB21_GOSHI	chlo	368.78	16.98%	[Uniprot] SWISS-PROT45:Chloroplast thylakoid membrane.
5	AKT2_ARATH	plas	386.223	7.48%	[Uniprot] SWISS-PROT45:Integral membrane protein.
6	At4g08920.1	nucl	388.277	7.64%	[Arath]
7	CHMO_SPIOL	chlo	389.926	12.30%	[Uniprot] SWISS-PROT45:Chloroplast stroma.
8	ISPF_CATRO	chlo	391.167	12.71%	[Uniprot] SWISS-PROT45:Chloroplast.
9	CB24_LYCES	chlo	393.657	16.60%	[Uniprot] SWISS-PROT45:Chloroplast thylakoid membrane.
10	MYRO_BRANA	vacu	394.687	8.58%	[Uniprot] SWISS-PROT45:Vacuolar.
11	UCRB_TOBAC	chlo	399.487	13.16%	[Uniprot] SWISS-PROT45:Chloroplast thylakoid.
12	CKX4_ARATH	extr	403.08	9.73%	[Uniprot] SWISS-PROT45:Extracellular.
13	UCRA_TOBAC	chlo	404.234	12.72%	[Uniprot] SWISS-PROT45:Chloroplast thylakoid.
14	MYR3_SINAL	vacu	405.487	8.64%	[Uniprot] SWISS-PROT45:Vacuolar.

SlHVA22k WoLFPSORT prediction nucl: 13, pero: 1

PSORT features and traditional PSORTII prediction

14 Nearest Neighbors

No.	id	site	distance	identity	comments
1	At2g32950.1	nucl	288.734	13.78%	[Arath]
2	TFB2_ARATH	nucl	327.372	14.13%	[Uniprot] SWISS-PROT45:Nuclear.
3	TGAA_TOBAC	nucl	327.572	11.63%	[Uniprot] SWISS-PROT45:Nuclear.
4	TF2B_ORYSA	nucl	351.031	13.06%	[Uniprot] SWISS-PROT45:Nuclear.
5	TF2B_SOYBN	nucl	353.896	11.45%	[Uniprot] SWISS-PROT45:Nuclear.
6	HSF8_LYCES	nucl	355.066	9.64%	[Uniprot] SWISS-PROT45:Nuclear.
7	TT1_ARATH	nucl	358.463	15.21%	[Uniprot] SWISS-PROT45:Nuclear.
8	PROL_ARATH	nucl	360.12	14.11%	[Uniprot] SWISS-PROT45:Nuclear.
9	PRH_PETCR	nucl	370.796	11.67%	[Uniprot] SWISS-PROT45:Nuclear.
10	HX1A_MAIZE	nucl	378.387	12.78%	[Uniprot] SWISS-PROT45:Nuclear.
11	At2g35670.1	nucl	384.704	13.27%	[Arath]
12	TGA7_ARATH	nucl	387.892	11.09%	[Uniprot] SWISS-PROT45:Nuclear.
13	AG_LYCES	nucl	391.01	11.81%	[Uniprot] SWISS-PROT45:Nuclear.
14	At1g79810.1	pero	394.711	13.77%	[Arath]

SlHVA22l WoLFPSORT prediction E.R.: 6.5, E.R._plas: 5, cyto: 3, plas: 2.5, nucl: 1, mito: 1

PSORT features and traditional PSORTII prediction

14 Nearest Neighbors

No.	id	site	distance	identity	comments
1	At2g38960.1	E.R.	304.311	12.71%	[Arath] sequence changed in 2004
2	NCPR_PHAAU	E.R.	306.133	9.86%	[Uniprot] SWISS-PROT45:Endoplasmic reticulum. Anchored to the ER membrane by its N-terminal hydrophobic region.
3	At5g53470.1	plas	310.007	11.54%	[Arath]
4	At5g17020.1	nucl	323.072	6.14%	[Arath]
5	At4g24520.1	E.R.	324.888	7.66%	[Arath]
6	At4g27780.1	E.R._plas	339.809	12.99%	[Arath]
7	F16Q_BRANA	cyto	342.847	15.93%	[Uniprot] SWISS-PROT45:Cytoplasmic.
8	At5g61900.1	plas	352.559	9.69%	[Arath]
9	MAOX_MESCR	cyto	354.851	9.06%	[Uniprot] SWISS-PROT45:Cytoplasmic.
10	At1g20330.1	E.R.	359.631	10.53%	[Arath]
11	At3g30775.1	mito	368.086	11.60%	[Arath]
12	F16Q_ORYSA	cyto	378.832	15.34%	[Uniprot] SWISS-PROT45:Cytoplasmic.

13	FD3E_PHAAU	E.R.	386.398	15.79%	[Uniprot] SWISS-PROT45:Endoplasmic reticulum.
14	At1g72280.1	E.R.	389.699	11.94%	[Arath]

The top two most similar predicted localization sites are highlighted in green.

Table S12. List of the HVA22 protein sequences used for phylogenetic analysis

Name	Accession	Sequence
CreHVA22a	Cre01.g027900	MIISAPICRVLMVVLGCAFPAYMTHKAVSSGNTQQLRHWCIYWLLMGFFMCVEWLADLSIFWLPLYYEAKLLFVVALWHPRTHAAITLYDTYILPLLRQHEATIDRHLSETRARLADAVTSQVNAAQGYVNNNAGAILQGLRTFSEQKPGAAPGSFSGQFTDLNHAKSQ
CreHVA22b	Cre03.g176930	MSVLEGVAHAVVQWVPLYFELKLVFVLWMTLPQTQGARMIYEGILAPRLAAWERRLRGGSAGNGRGAAAAAKAGSERDEPAGAIAAAILQNDEDRGEGGSSKVKASLRNLFHRTHKSEGAEEAGGVRGSSGGGGGGGGTAVGEGQEVGGVGGGGGVRLYSPLSAHLQQS
CreHVA22c	Cre03.g177500	MVLSFGLTYYVCIFAELLFPTYASLKAIQSPGKLDDTQWLTYWVVYAFISTFESVGSIILQWIPLYYEIKLLFVLWMIAPQTQGARKIYEDHIMPLLKKYGDKIDPVFARAEQALESQYVNHLAKYVDKHGPAVLDQALAQAQKHGTVVAGMAQAAYQEQLKKAASGQPGAAAFSGAH
CreHVA22d	Cre17.g696850	MAEQVAELASGFLSFANQVAAPFLKAALATGTAAPVAVAAPTPVPVAASLGLKVLLEPQMGGVIFRAASNTVATIYPVYASAKAIESKDTADDVQWLTYWTLYGSMILAEHLADQALGKVPFYYHAKFAALLWLQLPQTRGAAYLYNRYYKPAMAQYGPHIDAVLSKGHNLLINLYAMYKVPIEAAVALGLQAWKSLLAAVKALSEDKKAVNKSDAVKPHAA
PpHVA22a	Pp3c1_21270	MMGSFITRAIIMVLGYIYPAYECYKIVERKRPDPEYLRFWCQYWVIIAVVTVVERFADALISWVPLYSEAKLAFIIYLWYPQTMGTTYVYNMLLRPLVAQHELEIDRNLNELRTRAGDVVLQWWQRGSVHAQARFSELLQFLAMQSNGSQQRSFPNQGVPQGPARAPAQGHSREYPQGNSPLYPQPAAGFPIDSSYPPPEVRSHHETAPYPSSQGAQAGQELYPPIPPYSQPRRPYRSALSDGEGNFDIPEVKHRVPFSRMG
PpHVA22b	Pp3c2_21660	MAAAVSALGGELGLRLLLTPTASHVIVRTACTVVGIGFPIYSTHKAIENRNHSEQEEWLVYWAAYGCFSVAEVFSDKLLSWCPFYYHAKFVFLVWLQLPKNYGARHLYTSLLRPLLLKHHARLDRIVDGTRNEMHKFVMNHQSEVQAVQRMLKKFSVLVYQAAQDALQSARAIEGGSNPVGSRSSQESVTSPPPSPPSDSNYDETADDSGWIYTDHPRQSDQ
PpHVA22c	Pp3c3_14030	MGLLWPLSLRIYSLSGPAMMLIYPLYASIMAIESADKEDDQQWLTYWVLYSLVSLMEMAAGPVLAWIPFYSTLKLIVASWLVLPQFRGGIILYQKFVSPYLNATTGVSDQKLTEGQRKLLGSISSETQALVSTYIKENRSDAFDRIMEIAVKDLNDNKVEEVMDKESSSDPDSD

PpHVA22d	Pp3c4_23200	MGWLWALVLQGYAWSGPLMMLLYPLIASIMAIESPDMRDDRQWLTYWVLCSLALLLEIALAPVIVWIPFYSTIKLVIASWLVLPQFRGGTFLYENFVRPHFNDAAAAATNAQDPQQEWLERSVSPETQASVARFVEKHGSPGFEALMRAALVHPKLNSVDGMKERGLVDENAS
PpHVA22e	Pp3c5_7630	MMGSFITRGVIMLLGYVYPAYECFKVVERNRPDLEHLRFWCQYWIIIASLTVFERVGDIFVSWVPMYNEAKLAFIIYLWYPKTLGTTYVYSSFLRPFVVKHESEIDHQLNELTTRAGDLAFFWWQRGSAYIQAQVYQILAYVASQSNRTQQGTVVRQVPPAARQASIFRPNAQIPRGPPRDPPQGDTQVHPPPVGRFPGHTPGGAGYPALNVEELHPPAALQAGTTLYPPVAGEAGPDLYPPPPGPNFTGSAHRRRGRGAGTESAALLSENSDSDYDVVEHSAENCPESRPESQLEKKSQQSHPSIGEEGVPLKVYNTRNRLRSGGSRSLSEWLPWLGSTPSGKDD
PpHVA22f	Pp3c6_23420	MVKTKVPGVFDCFEEGLRYQLFKMMGSFITRGIIMLLGYVYPAYECFKVVEKNRPNLEHLRFWCQYWIIIAALTVFERLGEIMISWVPMYNEAKLAFIIYLWYPKTLGTSYVYFTFLRPFVVKHESEIDRQLNELTTRAGDLAFFWWHRSSGYIQAQFYQLLAYVASQSNRTQQGTDSRQIPQPSRQPPRPTSQIPHHDGPQGGPQVCPPPPSEGLPESAPGGAGYPPVNVEGPHPPPAAPLIGSGLYPPVAGQAGQGPYPPPPGPHSTGSVYRRRGSGTGTGPANLLSEDSDSDSDSDYDIVEHSAENRPEGRPEGRLESKPLQANPSTGVHDVPLKVYNTRNRLRSSGSR
PpHVA22g	Pp3c10_4040	MAVGYVYPAYECYKIVERPMPDLEHLRFWCQYWMIIAVVTVIERLADIVVSWVPMYSEAKLAFIIYLWYPKTMGTTYVYSTLLRPFVVQYESEIDHNLNEVRTRAGDIALLWWQKGSMYAQARFYELLQFLASQSNKVQQNPSGAPSRTPAQGLSRGLPQGVPQSYPPPGASGSETAPYPPQEAQAGQGQYPPPPSSSAGAHIRRQSGSRGVPVGDSDHGVVEKHSEKRTSYRLEDRSGTSVGGQNPPPRVQNTKTRPQTDSPWSISAWIPSLLGTTPSKKVD
PpHVA22h	Pp3c13_20961	MGWFWSLVLKSNALAGPIVMLLYPLYASVMAIESPFKEDDQQWLTYWVLYSFVSLLEMAAAPVFAWIPLYSTIKLAVAAWLVLPQFRGGFILYEKYVRPNFQNVTGYSGAKLTESQRKFLATISPETRSSVAKYIEAIGPESFENLIQKELEESRKKRAEEHQQGAVENPHVE
PpHVA22i	Pp3c13_20970	MMLLYPLYASIMAIESHDKEDDQQWLTYWVLYSLVSLMEMAAGPVIAWIPFYSTFKLIIASWLVLPQFRGGIILYEKFVSPYLNAATGVTDQKLTDGQRKWLGSISPEAQASVAAFIKENGSSAFDAFMKSATGDVKQDKIEETVEKKEGEAAGSSDSD
PpHVA22j	Pp3c14_4540	MCIIFNSQKDLKRNLRPGWFVTVALDINCRKSRVEPLKMMGSFITRGIIMGLGYICPAYECYKVVDRPRADLENLRFWCQYWMIIAVVTVLERLGDIFISWMPMYCEAKLAFIIYLWYSKTMGTTCLYSKLLKPFVAKHESEIDHNLNELKTRAGEIALVWWQKWSIHVQARFHELLQFLARQSDRAQEGARGSSSRSHALNIPRGLTQGALLSHPPPPPPGITGSPAASYPSSQGSQAGQRIYPPPLSHSTGVHVQHQLRPLGIFDGEGDSDHDVVEYHLGERAEHQSEERLERLVPSAGGQNLPPGVQNTRIRLQTDSPGNLSIWLPAWLGSTAPKKVDLQEGSRRYFGASYSFQLI
PpHVA22k	Pp3c16_15400	MMVSFITRGIIMLLGYVYPAYACFKVVERDRPDLEHLQFWCQYWIIIAALTVFERLGDVLVSWVPMYSEAKLAFIVYLWHPNTLGTKYVYSTFLRPIVARHEAEIDHHLHELTTRGGDLAYFW

		WQKCSNYAQSRIYELFSYVASQPNRTQQGSIARQSRRRPNFQGPPRGPPRDPYQGGPQVYPPAPPAGYPGSTRAGYPPVNANTLEDSDSDYHVVEKIVESRPEGRPEGGMKSRSEQEVPPHVGNIRERVRADDSGGSRYLYGWLPGWFGTSSGKED
PpHVA22l	Pp3c26_3490	MGWFWTLVARSNAVAGPIVMLLYPLYASVMAIESPFKEDDQQWLTYWVLYSFVSLLEMAAAPVFAWIPLYSTIKLAIAAWLVLPQFRGGVILYEKFVKPHFYAALGGSADSSSLSSGQRKWLGSIKPETRTAIAKYVQVKGPEAFEKLVNSELVKSISQKPRVEENPHTE
PpHVA22m	Pp3c26_3510	MGWLSVAVLRGYALAGPVMMLLYPLYASIIAIESADKEDDQQWLTYWVLYSVVSLLEMAAGPVIAWIPFYSTTKLVIASWLVLPQFRGGIILYEKFVSPYLNAATGQTDQKLTHSQRKVLGSISPDAQASVAAYIKENGPGSFDELMRRETANMHDEKVAEEKKHDGYSSSDPE
AmtrHVA22a	AMTR_s00010p00257430	MGLGAFLKVVAQNIDVLAGPIVTLVYPLYASVKAIESPSRVDDQQWLTYWVLYSMITLFEMTFYKAIAWFPFWSYAKLIAICWLVLPYFNGAAYVYEHYIRPCFVNPRQVNIWFFPGKKNALNNISDDARLALEKYLAENGPESFEKLLRKAEMEIKPNRSSLSIFDENYRY
AmtrHVA22b	AMTR_s00016p00231510	MLLYPLYASVRAIESPSKLDDEQWLAYWILYSFLTLMEMVAAPVLSWIPIWYQLKVAFVAWLVLPQFRGASFIYERFVREQLRKYDKAMGKGHRISPGKKNKFVDFVSPKAGEHEAY
AmtrHVA22c	AMTR_s00021p00253210	MGVLGAIARNLDTVVGPMVMLIYPLYASIRAIESPSALDDQQWLTYWVLYSLITLFELSCWRALAWFPLWPYMKLIFSLWLVLPIFNGAAYVYENVVKVHIPNLIKLNPDLTSSERRAIQMMTPEARGSVERFIAKYGSDAFEKVVKAAEKEAKKR
AmtrHVA22d	AMTR_s00030p00233590	MFRVREDGITQQRIVLGYAYPAYECFKCVEENKTDVDQLRFWCQYWIIVAMLTVFERAGDDFVSWLPMYGEAKVALFVYLWHSKTKGTTYVYQTFVRPYVSKHEGEFDRHLVEFRARAGGLFLMYWQCVARYGHARFFEILEYLSSQSSASNHPQNNKSEAQPSQAEREEILKAAQVAIPHPPPPEKQSQGRKMTGFQKTKRV
AmtrHVA22e	AMTR_s00238p00013230	LALGYTYPAYACFKTVEKNKPEIDQLCFWCQYWIIVAVLTVFERVGDTFVSWVPMYSEAKVAFLVYLWYPKTLGTSYVYETFLRPYVAKHETEIDRNLLELRARAGDIVVQFWQKTTSYGQTRIFEILQYVASQSSRPAPAQVSVDLGK
AmtrHVA22f	AMTR_s00280p00015410	LALGYTYPAYACFKTVEKNKPEIDQLRFWCQYWIIVAMLTVFERVGDTFVSWVPMYSEAKVAFLVYLWYPKTLGTSYVYETFLRPYVAKHETEIDRNLLELRARAGDIVVQFWQKATSYGQTRIFEILQYVASQSSRPAPAQ
AmtrHVA22g	AMTR_s00059p00092060	MALLGPQVAGEVGLRLLLCPFGSNIVVRTACCSVGIALPVYSTFKAIENKDQKEQEKWLLYWAVYGSFSLVEAFADKILYWFPLYYHMKFAFLVWLQLPTVEGAKQLYLKYLRPFLLKYQRRLDQILVVVSGEMRKFISAHQGEIQFMGAMVQKSVGAGFLVESHMLHSLSALNYTRERSSRGLGRRLNELRLHHGNSRTWPRALRLQRSRAGGREESCGRELGGLVLERRDRYRP
AtHVA22a	AT1G19950	MIGSFLTRGLVMVFGYAYPAYECYKAVEKNKPEMQQLRFWCQYWILVAALTIFERVGDALASWVPLYCEAKLAFFIYLWFPKTRGTTYVYDSFFQPYVAKHENEIDRSLIELRTKAGDLAVIYCRKAVSYGQTRIVEILHFVALQSTPKPKPKEKKQAAAPEEEEQKQPDLKATSQAASSNPQVRL

		QSKKPQLVTKEPISPKPLSSPRKQQQLQTETKEAKASVSQTKLTTLTPPGPPPPPPPPPPSPTTA<br>AKRNADPAQPSPTEAEEASQTVAALPEPASEIQRASSSKETIMEETLRITRGSLRKARSAGAP<br>R
AtHVA22b	AT1G69700	MPSNSGDDNVLQVLIKNFDVLALPLVTLVYPLYASVKAIETRSLPEDEQWLTYWVLYALISLF<br>ELTFSKPLEWFPIWPYMKLFGICWLVLPQFNGAEHIYKHFIRPFYRDPQRATTKIWYVPHKK<br>FNFFPKRDDDDILTAAEKYMEQHGTEAFERMIVKKDSYERGRSSRGINNHMIFDD
AtHVA22c	AT1G74520	MGSGAGNFLKVLLRNFDVLAGPVVSLVYPLYASVQAIETQSHADDKQWLTYWVLYSLLTLI<br>ELTFAKLIEWLPIWSYMKLILTCWLVIPYFSGAAYVYEHFVRPVFVNPRSINIWYVPKKMDIFR<br>KPDDVLTAAEKYIAENGPDAFEKILSRADKSKRYNKHEYESYETMYGEGYQY
AtHVA22d	AT1G75700	MIGSFLTRGLLMVFGYAYPAYECFKTVELNKPEIQQLQFWCQYWIIVAALTIFERIGDALVSW<br>LPMYSEAKLAFFIYLWFPKTKGTTYVYDSFFRPYIAKHENEIDRNLVKVKTRAKDMAMIYLQ<br>KAINQGQTKFFEILQYITEQSTPKSKAEEKKETTIPKLDDPILKVKENEVTK
AtHVA22e	AT2G36020	MLGDFIIRLLVLILGYTYPAFECFKTVEKNKVDIEELRFWCQYWILLALISSFERVGDFFISWLP<br>LYGEMKVVFFVYLWYPKTKGTRHVYETLLKPYMAQHETEIDRKIMELRARAWDFFIFYFNN<br>FAQAGQSTLIQGFQYVLAQSVRFSAAAANQPPTERNVNMNAQSPVEMDNDPPSPRAPRPL<br>NKSLSALRSLEKQTSRGRKWPPPTPPPTPGRDSAGTFNGDDGVNIPDTIPGSPLTDARAKLR<br>RSNSRTQPAA
AtHVA22f	AT2G42820	MGFIIAIAKRFDALVGPGVMLLYPLYASFRAIESPTMLDDQQWLTYWIIYSLITIFELSVWRVL<br>AWLPFWPYLKLLFCMWLVLPMFSGAAYIYSNFVRQYVKIGMNVGGGTNYTDEQRRVLQM<br>MSLDARKSVQDYVDRFGWDSVEKAIKAAEKETRKH
AtHVA22g	AT4G24960	MLLYPLYASVIAMESTTKVDDEQWLAYWIIYSFLSLTELILQSLIEWIPIWYTVKLVFVAWLVLP<br>QFQGAAFIYNRVVREQFKKHGVLRSTHSKPTKPNILHSIFPHRSVPNSLTRDTRLTVTESEGW<br>SRQKKPD
AtHVA22h	AT4G36720	MSGFASQIPSMALLGSGLTGEVGLRVLFSPLSSNIVLRTACCSIGIGLPVYSTFKAIESGDENEQ<br>QKMLIYWAAYGSFSLVEVFTDKIISWFPLYYHVKFAFLVWLQLPTVEGSKQIYNNQIRPFLLR<br>HQARVDQLVDGVYGEMVKVVRSHQGEIRFVRAMIAKILGSVNEDAPRLGEIANGSPVSET<br>NSDSESDSNHED
AtHVA22i	AT5G42560	MVLGYAYPAYECYKTVEKNRPEIEQLRFWCQYWILVACLTVFERVGDAFVSWVPMYSEAKL<br>AFFIYLWYPKTRGTTYVYESFFRPYLSQHENDIDHSLLELRTRAGDMAVIYWQRVASYGQTRI<br>LEILQYVAAQSTPRPQPPQKRGGRANQAPAKPKKAPVPQSEPEEVSLSSSSSSSSSENEGNEP<br>TRKVSGPSRPRPTVTSVPAADPKNAGTTQIAQKSVASPIVNPPQSTTQVEPMQIEEVEGEAES<br>GNENPNPEGPKETVMEETIRMTRGRLRKTRSEESR
AtHVA22j	AT5G50720	MTKLWTSLSALHSLAGPVVMLLYPLYASVIAIESPSKVDDEQWLAYWILYSFLTLSELILQSLL<br>EWIPIWYTAKLVFVAWLVLPQFRGAAFIYNKVVREQFKKYGILKPKVEHQAE

AtHVA22k	AT5G62490	MSSGIGSLVKVIFKNFDVIAGPVISLVYPLYASVRAIESRSHGDDKQWLTYWALYSLIKLFELTFFRLLEWIPLYPYAKLALTSWLVLPGMNGAAYLYEHYVRSFLLSPHTVNVWYVPAKKDDDLGATAGKFTPVNDSGAPQEKIVSSVDTSAKYVGHSAFDDAYIY
SlHVA22a	Solyc01g007780	MLGELITSSLVLVLGYAYPAFECYKTVENSRVEIEELRFWCQYWIIVALLRIFESFGDVFMTWLPMYSEAKLALIIYLWYPKTKGTRYIYDTLLKPYVSRHEPDIDRGFLEFRTRAFDLAIYYWQNCTELGQAKFFQLLDFASPSRRDSHLISEQRNGTDHSRRSPPPSAPPSPSSSSSFGFFRRSKPSSDRRQPPPPSPRSDRRQPQSPRSYSSHRSIY
SlHVA22b	Solyc03g097420	MSKLWALLTHLHALAGPVVMLLYPLYASVIAIESTSKLDDEQWLAYWILYSFLTLMEMLLQPILQWIPIWYEVKLGMVAWLVLPQFRGAAFIYNKFVREKLIKKYGSSYIQHKSQSPDGKTKNKIVDFITLKKGDH
SlHVA22c	Solyc03g110920	MAGMAMFASNLSSEMGLKLLLSPLNTNVIVRTACCSVGIVLPVYSTFKAIETGNRNEQHKWLLYWAAYGSFSIVEAFTDKFLYWFPLYYHVKLAFLVWLQLPSAEGAKQLYTNHLRPFLMKHQARLDHILELFHGELGKFISTHQAEIQFAKVLLGKTLLSVGNILQQPAQGRVVGTIEGPAEQVETSESEDES
SlHVA22d	Solyc03g116350	MGGSGGGIVSLLKVIISNFDVLAGPVVSLVYPLYASIRAIETKSPVDDQQWLTYWILYSMITLFELTFAKLIEWLPFWSYAKLIATCWLVIPYFNGAAYVYEHYIRTYIVQRKAVNIWYVPRKKDVFSKPDDILTAAEKYIKEHGTQAFEEMIHKADGERKTHTSNYVFYDDDYRY
SlHVA22e	Solyc04g014420	MLCRYASIVALESTSKLDDEQWLAYWILYSFLTLVEMLFQPILNWIPIWYDVKLIGVAWLVLPQFRGATFIYETYVREKLIMNKYGDQGKSKKKSVHFMTPEKGEDEAYD
SlHVA22f	Solyc04g076610	MGLISFLLISAKILISCSFWIFCRPSVTLLCPLYASIRAIESDKESSYEKCLQYWVLFGLTTVLELTLAKPLTGFSFWHYAKGLASLLLVLPQFGVASYVYMNFVKPCLSPNPHIEYVTEKNKSMPMELSCADSSLLMSHPRRVLQKYTTFGESDTQLLTCTKSSSNIAMKCSNYVDAAPRHVEKYKKDNLQSLVIYEAESSPKCDRTICPKKVQMDWSCPLCLVSVSSEKCLKQHIKGRKHKLKEDEQREHEMIMASKEAKLASKLEGNYLKDLLESLNQIKSLKFVELRGFNLPIRRPLRCCTWKKPKPGWTKLNTDGSIDRKRAGLGGLLRDYEGAAICACVSEVTCDDIFLVELLAIWRGLMLAVSIGIKMIWVESDSMGAVKAINKEQPHNQKAASCLQHIWKMLNKFQKYQVTHSWRETNRAADYLSKMEISGSDIVMWPREFHGPLCKIIAEDAQGSLYIRR
SlHVA22g	Solyc04g081340	MIGSFITRVLVMVFGYAYPAYECFKTVEMNKPDIQQLRFWCQYWILVAMLTVCERFGDAFISWVPMYSEAKLAFVIYLWCPKTKGTTYVYDAFFKPVILRHEPEIDKSLLELRTRAGDMFFLYWQKAASYGQTRVFDILQYIASQSNPPPPTQTHRQSSRGRQPTASLNRRSSASATQVQAEEQAPRASSESSSEDEADSAEEAGSSKGPPPASTAAANAQKTTPSKSLVSTVAASLNTQKASPSKSLAEITKPSTSVETRVVQIDSVPPSATESVNPPAETALEEAVRVTRARSRKTRTSNNP
SlHVA22h	Solyc05g007300	MGSDNNVLAVIAKNIDVLALPLISLVYPLYTSIKAIETKSRADDRQWLTYWVLYSLITLFELSFSKLIEWFPIWSYAKLAAICWLVLPYFNGACYVYENFIRPFYRNPQVKIWYVPLKKDIFSKPDD

		VLTAAEKYIEEHGPQAFERLLAKADRDARTRRNNYMTFDDDYRY
SlHVA22i	Solyc06g072680	MGKFLTLMTHLHTLAGPSVMLLYPLYASVVAIESTSKLDDEQWLAYWILYSFLTLMEMLLQP<br>MLQWIPIWYDLKLAMVAWLVLPQFRGAAFIYEKFVREKLINKYGARYFRDKSPPPVAVSPA<br>KVSVKTSEDRTD
SlHVA22j	Solyc10g007820	MGAIGTIAKSLNALIGPGVMLLYPLYSSMRAIESPSPLDDQQWLTYWVLYSFITLFELSCWKV<br>LQWLPFWPFIKLVCCMWLVLPIFNGAAYIHENFIRKHVKVGSHVSSNYPQNQRKALQMMS<br>LDARKSVEKYIEKYGPDAFDKVVRAAEREAKKH
SlHVA22k	Solyc10g047670	MDFLRFSLHFIDCLAWPVVALGYPICASIRAIETGSKYHMRKLVIYWTIFSFFSLFQHLFDKLI<br>QWVPLWPYIKLITICWLVIPRFNGACYLYEVLVRPCLLVKWHDSISQFNSSCYVYLRLLCLCL<br>SVNLRTVADWFNKPMEDRSLKNETFLSVVERYLEENGSDALEKLIANKYKDYSSKHHAEEI<br>KPTDTSDEAGRITPNQTQCEGSGPVWEDITVMEHMAKHEAAEPKQVKSVKENPIIIEQKMT<br>GLQVKELVVPADAEEIKLPEVISPNRVQTEWTCALCQMTTTSEQNMKSHLNGRKHKSKYE<br>ALKICEQTPKSNGSLPVPTKSNQLNLEQVKHAAAAQPFHSTNEAAEPKEVKSVKEHPIQIE<br>KKTTGVQTKDTAFPAEAKEIKLPEIDSVKNVKTEWTCAVCQVTTTSKGDLKCHLLGTRHRI<br>KCEELKRTTKTERNPPSTSNMPELKQEQVKHALAAQYKNSTNKKLKENVQLGATTGQHQ<br>RQTQVKNAGGATHNSKLWCSFCDIRCPDEIAMAAHLNGKKHLAKLQERMSLTTGTWALN<br>YANMQFYQGI
SlHVA22l	Solyc10g051300	MDPEVGLRLLFSPLASNIVVRTACCSVGVVLPVYSTFKAIEGRDENEQRKWLLYWAAYGSFS<br>VVELFTDKFLYWFPLYYHMKFAFLVWLQLPTTDGARQMYMTHLRPFLLKHQARLDQVVGF<br>LYGQMSKFVSMHQAEIKFVRALLMRTFVSANQFASGFIHPERRHVSGAIEERRQQYDTSDSD<br>DEE
SlHVA22m	Solyc10g082040	MLGNFITSVLILVLGYAYPAFECFKTIEKNKVEIHELRFWCQYWIIVGALRIFESFGDLFLSWLP<br>MYGEAKLALFIYLWYPKIKGTSHIYDTILKPFVAKYETDIDRSLMEFRAKAWDLAIYYWQNC<br>TEIGQAKFLQMLEYIASQSKRGTIHPKSEKQDETTHRSSSPTIDHKDFYGARAKLRRSNKGD
SlHVA22n	Solyc11g010930	MGHFWTLICYLHTLAGPVTMLLYPLYASVVAIETSDKLDDEQWLAYWIFYSFLTLMEMVLQ<br>HVLEWIPIWYDVKLIFVAWLVFPHFRGAAFIYDKFVREKIIKRYRESSSSSPQHINKSPKAKSK<br>TKFVDFITPKKGEHEAY
SlHVA22o	Solyc12g089290	MQCNAGRFAGFKMIGSFLTRGLVMVFGYAYPAYECFKTVEMNKPDIQELRFWCQYWILIAL<br>LTVCERVGDAFVSWVPMYSEAKLAFIIYLWCPKTKGTTYVYDSFFRPVVLKHETEIDRNLLEL<br>RTRAGDMACLYWQNSASYVQTRFFDVLQFIASQSTPRPTQPQKQSSRGRQRTVTPPKCRSA<br>APATKVQTEKQAPPASTESSSEKEADIKEKVKPSQPPPVATPASSASSSNAQKTTPAEAHAQI<br>TKASSLSKTEVMQVDQVSSSDNKSAKPLVDTVMEDNITAKPVDTVMVEAVRVTRARSRRTR<br>PAPNP
StHVA22a	PGSC0003DMG40	MGGSGGGIVSLLKVIISNFDVLAGPVVSLVYPLYASIRAIETKSPVDDQQWLTYWILYSMITLF

	0000600	ELTFAKLIEWLPFWSYAKLIATCWLVIPYFNGAAYVYEHYIRTYIVQRKAVNIWYVPRKKDVFSKPDDILTAAEKYIKEHGTQAFEEMIHKADGERKTHTSNYVFYDDDYRY
StHVA22b	PGSC0003DMG400003727	MIGSFITRVLVMVFGYAYPAYECFKTVEMNKPDIQQLRFWCQYWILVAMLTICERFGDAFISWVPMYSEAKLAFVIYLWCPKTKGTTYVYDAFFKPVILRHEPEIDKNLLELRTRAGDMFFLYWQKAASYGQTRVFDILQYIASQSNPPPPTQSLHAYESIQLCCDATPQSPSLTVRADLKPQRQSSRGRQPTASLNRRSSASATQVQAEEQAPRASSESSSEDEADSAEEAGSSKGPPPASTAAANAQKTTPSKSLVSTVAASLNTQKASPSRALAEIMKPSTSVETQVVQIDSVPSSATESVNPPTETALDEAVRVTRARSRKTRTSNNP
StHVA22c	PGSC0003DMG400004398	MFTNSFRYASIRAIQTDSKYHMRKLLTYWIIFSLFHHIFDKLIQWVPLWPYIKLITICWLVIPQFDGACYLYQKLIHPCLLVKLHDVITQFYGFCYVYQRFLYVCLSVNLQIVADWLNKPMEDPSLKNVSFLTTAERYLEENGSDALLKLIANKCKDNNLNHHAEEIKTTDTSVEAGTLIPNQTLCEGACPVWQNIKPMEHMAKYEAAQPKQVVIFNP
StHVA22d	PGSC0003DMG400009332	MGLISFLLISAKILLCCSFWIFCRPSVTLLCPLYASIRAIESDKESSYQKCLQYWVLFGLTTVLELTLAKPLTGFSFWHYAKGLASLLLVLPQFGVASYVYMNFVKPCLSPNPLIEYVTEKNECMPMERSCADSSLSMPHPRRILQKYTTFGESDTQLLTCFKSPSNIVMECSDYVDAAPRHVKQDKKDNLQSFVIYEAESSPKCDRTIYPKKVQKNWSCPLCLVSVSNEKCLKQHIKGRKHKLKEDEEREHEMIMTSKEAKLASKLEGNYLKDLLESLNQIKSLKFVELRGFNLPIRRPLRCCTWKKPKPGWTKLNTDGSIDRKRAGLGGLLRDDEGVAICACVSEVTCGDIFLVELLAIWRGLMLAVSIGIKVIWVESDSMSAVKAINKEQPHNQKAASCLQHIWKILNKFQKYQVTHSWRETNRAADYLSKMEISGSDIVMWPRDFHSPLCKIIAEDAQGSLYIRR
StHVA22e	PGSC0003DMG400010683	MDLVSVTIACINLLAWPMLALGYPLFVTIRAIEAGCVYDKRKVATYWLIFSLIYLFENIFAKFLEWVPLWSYLRLVFMYWLVIPQLNGAFYLYQNLVHPYLQFKLPNAIIQLCAEWLKILKNHSVSINNETFLAVAESCLDENDSLIASKPECNEGGQILGDDIQEMECTAKCNAVESNQIENISTSAIQVISKPVIPAVAILPDTSSTLAVFQTRWTCEICQVTVSSELTLQSHLRGRKHRAEAMLWCTFCNLRVSGEIDMIAHLKGKRHLAKLQETLTNSGAS
StHVA22f	PGSC0003DMG401015201	MQMGLKLLLSPLNTNVIVRTACCSVGIVLPVYSTFKAIETGNRNEQHKWLLYWAGDDAFQNFTNFFCFANLEIAFFSTRY
StHVA22g	PGSC0003DMG400016187	MLLYPLYASVVAIETSDKLDDEQWLAYWIFYSFLTLMEMVLQHVLEWIPIWYDVKLIFVAWLVFPQFRGAAFIYDKFVREKIIKRYRESSSSSPQHNNKSPKAKSKTKFVDFITPKKGEHEAY
StHVA22h	PGSC0003DMG400016386	MLGELITSSLVLVLGYAYPAFECYKTIEKNRVEIEELRFWCQYWIIVALLRIFESIGDVFMTWLPMYSEAKLALIIYLWYPKTKGTGYIYDTLLKPYVSRHEPDIDRGFLEFRTRAFDLAIYYWQNCTELGQAKFFQLLDFASPSRKDSQLSSEVLFSHTFSPYVFSSKQSLTEMQKKTVYNRTL
StHVA22i	PGSC0003DMG402023061	MGSDNNVLAVIAKNIDVLALPLISLVYPLYTSIKAIETKSRADDRQWLTYWVLYSLITLFELSFSKLIEWFPIWSYAKLGAICWLVLPYFNGACYVYENFIRPFYRNPQVKIWYVPLKKDIFSKPDD

		VLTAAEKYIEEHGPQAFERLLAKQADRDARTRRNNYMTFDDDYRY
StHVA22j	PGSC0003DMG40<br>0026482	VFCRYASVVAIESTSKLDDEQWLAYWILYSFLTLMEMLLQPILQWIPIWYEVKLGMVAWLVL<br>PQFRGAAFIYNKFVREKLIKKYGSSYIHQKSHSPDGKTKNKIVDYITLKKVKL
StHVA22k	PGSC0003DMG40<br>0026483	MSKLWALLTHLHALAGPVVMLLYPLYASVVAIESTSKLDDEQWLAYWILYSFLTLMEMLLQP<br>ILQCDCGMSFFEFLSDEINMPSNDFRSDYLRTRYATLLLKYGLNKAETGYVSENDDPTRSKSD<br>YTPPTKDDLVNVE
StHVA22l	PGSC0003DMG40<br>1026939	MFDILFCDDERYASVVAIESTSKLDDEQWLAYWILYSFLTLMEMLLQPMLQWIPIWYDLKLA<br>LVAWLVLPQFRGAAFIYEKFVREKLITKYGARYFRDKSSPPKVSVKPSEDWTD
StHVA22m	PGSC0003DMG40<br>0029015	MDFLRFALPLIDCLAWPVVALGYPICASIRAIETGSKYHMRKLVIYWTIFSFISLFQHLFDKFIQ<br>WVPLWPHIKLITICWLVIPRFNGACYLYEVLVRPCLLVKWHDSISQLNSSCYVYLRLSCLCLS<br>VNLQTVADWFNKPMEDQSLKNETFLSVVEGYLEENGSDALEKHIANKYKDYSLKHHAEEI<br>KPTDTSDEAGRITPNQTQCEGGGPVWEDITVMEHMAKHEAAEPKQVKSLKENPIIIEKKTK<br>GLQVRELVVPADAEEIKLPEVASSEKVQTEWTCALCEMTTTSEQNMKSHLNGRKHKAKYE<br>GLKTCEQTSKSDGSLPVPTKSNQLNLEQVKHASAAQPQHSANKAVEPKQVKSVKEHPIQIE<br>KKTTGVQIKDTAFPADAKEIKLPEINPLKNIQTEWTCAVCQVTTTSKHDLKCHLLGTRHRIK<br>CEELKRTAKTERNPPSTSNIPELKQKQVKHALAAQNKNSTNKKPKENVQLGATTRQHQRQ<br>TQVKNAGGATHNSKLWCSFCDIRCPDEIAMAAHLNGKKHLAKLQERMSFTSGTSWD*
StHVA22n	PGSC0003DMG40<br>0029421	MVFGYAYPAYECFKTVEMNKPDIQELRFWCQYWILVVLLTVCERVGDAFVSWVPMYSEAK<br>LAFIIYLWCPKTKGTTYVYDSFFRPVVLKHETEIDRNLLELRTRAGDMACLYWQNSASYVQT<br>RFFDILQFIASQSTPRPTQPQKQSSRGRQRTVTPPKRRSAAPATKVQTEKQAPPSSTESSSEKE<br>ADTKEKVKPSQPPPVATPASSASSVNAQKMTTAEAHAQITKASSLSKTQMMQIDQVPSSDN<br>QSAKPLVDTVMEDSLTAKPVDTVMEEAVRVTRARSRRTRPAPNP
StHVA22o	PGSC0003DMG40<br>0030143	MGFLIFTLHIMEFLAWVPLWPYIKLITICWLVIPQFDGGCYLYQKLIHPCLLVKLHDAITQFYG<br>FCYVYQRFLHVCLSVNLQIVADWLNKPMEDPSLKSESFLDTAERYLDENGSDALLKLIANK<br>CKDNNLNHHAEEIKTTDTSVEAGTLIPNQVLQLLFRAPCTV
StHVA22p	PGSC0003DMG40<br>0033909	MDPEVGLRLLFSPLASNIVVRTACCSVGVVLPVYSTFKAIEGRDENEQRKWLLYWAAYGSFS<br>VVELFTDKFLHWFPLYYHMKFAFLVWLQLPTTDGARQMYMTHLRPFLLKHQARLDQVVG<br>FLYGQMSKFVSVHQAEIKFVRALLMRTFVSANQFASGFIHPERRHVSGAIEGRRQQIDTSDS<br>DDEE
CcHVA22a	Ciclev10029342m	MALSGEVGLRLLLCPIGSNIVVRTACCSVGTILPVYSTFKAIESKDENEKQKWLVYWAVYGSF<br>SIAEMFADKILCWFPLYHHVKFAFLVWLQLPSTNGAKYFYMSRLRPFLLRHQARLDQILESV<br>NGEMSQFVSDHQVEFRFVRTLFMKTVALVNQTVKEIIHPVPTQANRAIEGPPESIPDSQSDN<br>ED
CcHVA22b	Ciclev10033698m	MQNFACATLLHWLLLALNPYIKGSETNARPKKKVGLKSDFWGSSLRVPLFNFVKLARKNH

		FEALGPVNSIAPNTQKSQTNKGRHLKQLMGARGSNNNNFLQVVVNNFDVLALPVVTLVY<br>PLHASIKAIEARSASDDQQWLTYWVLYSMITLFELTFAKVLELITIWPYAKLIFSCWLVLPQFN<br>GAAYVYRHFVRPFYMNPQSASSKIWYVPRKKNIFRQQDDILTAAEKYMQEHGTESFERLIA<br>KTEREERSRKSNNYMIFDDDYRY
CcHVA22c	Ciclev10022553m	MGSGAGSFLKVLLKNFDVLAGPVVSLVYPLYASVRAIETKSPTDDRQWLTYWILYSMITLVEL<br>TFAKVIEWIPIWSYAKLIFTCWLVIPQFSGAAYVYEHYLRPFFLNPQTINIWYVPRKKDVFSRP<br>DDILTAAEKYMEENGTDAFEKLIHRADKSKRNYDHSTYEYDNVY
CcHVA22c*	Ciclev10022663m	MGVFFHLGHLLRYASVRAIETKSPTDDRQWLTYWILYSMITLVELTFAKVIEWIPIWSYAKLIF<br>TCWLVIPQFSGAAYVYEHYLRPFFLNPQTINIWYVPRKKDVFSRPDDILTAAEKYMEENGTD<br>AFEKLIHRADKSKRNYDHSTYEYDNVY
CcHVA22d	Ciclev10033011m	MSRFWNLIYGVHSLAGPVLMLLYPLYASVVAIESPSKVDDEQWLAYWILYSFLTLTEMVLQP<br>VLEWIPIWYSVKLVLAAWLVLPQFRGAAFIYERFVRQQIRQYRGGKDHHQHQHRKSSPTGT<br>GKGKNKFVDFIMPKKGEHEAY
CcHVA22e	Ciclev10032960m	MGVLAVIAKRLDALIGPGVMLLYPLYASLRAIESPSTLDDQQWLTYWIIYSFITLFELSCYKVL<br>AWLPFWAYMKLLFCMWLVLPMFHGAAYIYEKHIRRYVKIGGYVSPSSNYTADQRKVLQMM<br>SLDARKSVSQYVEKHGWEAVERAIKAAEKETKKR
CcHVA22f	Ciclev10006951m	YISLETLERPSAPDYRQWMTYWIILSLVTTFEMLCWKTLGWFPVPNFIWAYLKLLIFVWLALP<br>IFNGACFFYEKFIRVYYNNYVRRHLNNIIGV
OsHVA22a	LOC_Os01g52780	MRAVDASKCWPFGRGGGDGEPPPPLPPMEPPKRSRWWAHELAAERARQEARATGTEDAG<br>GGVAGGGGSGKGAKRKGSRGRVRAERARKWRRSLQFGLLSKRKEKTSSRLLHYVLHKQLL<br>SKHKGSTLRTQKEPSAQKSKHKGSTLRTQKELSAQKKFQNTHDCMSTHENNLNKQYIRGM<br>DPSTGMGSNLVRKEAANSSVNKQNIKASEPTIYPLNPGCELVKNVAYPPKDDIFGDLPLLESS<br>KVMFHSGVDELPTVIEDSFVTDQSGPEAISENVSLKLIPPSDMSVQTSSTLKDLVKKEGTPAK<br>KSICILRNDVKKNLPSSAEFDGLNHNGINMVKTCLSDTQLKSTDVPALSSYSNKGLKSGSSN<br>TAHTQQDCFSMNTNYCQEIRKPGTSIATSSVTVRTRTEAIESDRDMAVNSKKSTSISCALVPT<br>ECHISSEGSVLSSAISQGSASAATSADGMSSYKSIPSQDSIPTSGLNGTFASNLFRESRKSVDTC<br>TSLSKEDQCSWYSKLHPVCTPASIGSAFMKLPGLERIEISSCNIKTDENMSTNGRPANIVRCE<br>KQQAVIGVPNIMQGQRKTDFSDSQVQEKVLNGYLQQDVYHPCQPTVRLMGKTVSVCERS<br>KEHRVPTMGKGWSDSTIVEDHPSSTSCHFPQKRLFPCQDSVTPSVHVKGSLDILQRIPSVTLP<br>EARATIGNVQNRRLQPINTVSSSVRDCIWNSGSQSVRQAEIKRATTINVNSRARHINLHQPP<br>QEKYQKSTFSYDDPTSAPIYQSCQDLVAPVFEETKNRSLGWSLDDAIGPRILDFGSKVAGHGL<br>EMSTNESNCVRASSGPVPVLETRSIKPSSLSLAIYTLVQRLIEDIEKIQNKALHRSQQRIMGVL<br>GALARHMDALVGPGIMLLYPLYASMRAIESPSTLDDQQWLTYWVLYSLITLFELSCWKVLQ<br>WFPLWPYMKLLFCCWLVLPIFNGAAYIYETHVRRYFKIGQYVSPNYNERQRKALQMMSLD

		ARKSVERFIESHGPDALDKIIRAAEEEAKRA
OsHVA22b	LOC_Os02g02920	MYSEAKLAFIVYLWYPKTQGTSYVYESFFKPYIGKHEAEIDRNLLELRTRAGDMAVHYFQKI<br>ADYSHTRFYEILQYIASQSEAQRSRPQAQQHQQRPPPPRTRQVNPAPPPVPSPSAPPLPPQPP<br>PPRNQAQADKAPIPVAPPGAAVPPAQPQPQPPQAGAEAVTTESTEATQAANPPATTASNPH<br>QAPVIPDEETLIQEAIRMTRSRLRRRMGGA
OsHVA22c	LOC_Os02g51290	MGSGSGSFLKVVVKNLDVLAGPIVSLAYPLYASVRAIETKSAVDDQQWLTYWVLYSFITLFEL<br>TFSPVLEWLPLWSYAKLFFNCWLVLPYFNGAAHVYEHFVRPMVVNQQIVNIWYIPRKDESD<br>RPDDVISAAQRYIEQNGSRAFESLVNKVLSTYGSI
OsHVA22d	LOC_Os03g04030	MAGSFITGALMLILGYAYPAYDCYKTVELNKPEIEKLRFWCQYWILLAVLTVFDRVGDNFVS<br>WLPMYSEAKLAFVVFLWYPKTLGTAYVYESFFKPWIAKYEADIDHNLLELRTRACDMAVLY<br>FQKVSNYGQTRLYEILQYVASQSQTQTSRPQAREQQQRPPPAQTRQVNPAPQPVPAPSVPPL<br>PPQPTQAPSAPPRNQTQDTTPVPVPPPGAESLAQPQAHAGPPQANASDGPQNTEAMQIDP<br>SGPSTSNARQSSIPDEDTLIQEAIRMTRGRLRRRTAGSGPPPS
OsHVA22e	LOC_Os03g14610	MMGGFLSRVLLLAFGYAYPAYECYKTVELNKPEIEQLIFWCQYWILVALMTVMERFGDFTIS<br>WLPFYSEAKLMFFIYLWYPKTKGTTYIYGTFFRPYISQHENEIDRNLLELRARATDVVVLYFQ<br>KAATVGQNTFFDVLKYVASQSPSQRSSQQPSQEPQQPKQQQAPVQQQPTQKQAPTVLRRS<br>ASIAARQAAMAQQSQDAKTVPSSPKIKRQASTKAAPVASTKLTGAAAPSTPKSDADAPKK<br>NEAAPASLQVATPATKADVPASEPSAPLPEAEEADKMAIDEADDAVEGTEEGDPVPGETVE<br>ERPMEETIRVTRSKLRRRTASEDPAGN
OsHVA22f	LOC_Os04g33880	MSVEFLTKALTALFGYAMPALECFKAIEQRPGRTDHLRFWCQYWIILVILVIFDDIAGVLTSKI<br>PMYSELRLAFLVYLWYPQTRGTDIVYDTFLRPLVMQYQPNIEERLRYLRANAGDILIFYLKNF<br>TDRGYDLFLRGMEYIRSQTSRGSRTRVCYQLLSVPYRKVF
OsHVA22g	LOC_Os06g12220	MGSGSFLKLLANNFDVLAGPLVSLAYPLYASVRAIETKSPVDDQQWLTYWVLYSFITLFELTF<br>APVIEWLPFWSYAKLFFNCWLVLPCFHGAAYVYDHFVRPMFVNRQIVNVWYVPRKENLSK<br>PDDVLSAAERYIEQNGPEAFEKLISKSTRPSTSKRSTKQSILEEVESEHMARAERESWGENPFY<br>DKNYRC
OsHVA22h	LOC_Os07g38100	MALLAPAISGEVGLRLLLAPLSSTVVIRTASCAIGVGLPVYSTFRAIEKKDQKEKERWLLYWA<br>AYGSFSIAEIFADQILSSVPFYYHVKFAILVWLQFPSNSGAKHVYRRYMRPFFLKHQAKIDRIL<br>NILSKELNKFVSSHEDEIHFIEHMAARGASTANYIINGPEQSEAVRAAIEGPNSTTTEEAGTP<br>RNET
OsHVA22i	LOC_Os08g36440	MESPTKVDDEQWLAYWILYSFITLLEMVAEPVLYWIPVWYPVKVLFVAWLVLPQFKGASFIY<br>KKLVREQLRKYRARGGAAATVTAGEDHKVHIAKAEHDHGH
OsHVA22j	LOC_Os09g27730	MESTSKVDDEQWLVYWILYSLITLMEMALHKVLYWIPLWYEAKVLFVAWLVLPQFRGASFIY<br>DKFVREQLKKNRVKLHEHHGHGHGHADEHQSHVVRG

OsHVA22k	LOC_Os09g36340	MAVLLITRLLTLVLGYAYPAYDCYKTLELNKPQIDQLRFWCQYWILLAFLTTLETITYFTVSWL PMYGEAKLALVLYLWYPKTRGAKHVYESYLQPVLARHEADIDRGLLQLRASAKDATASHL QAAVSLGRACFAEVAGRVSSQLQAARSSGGGGRAGQADQLQKRQKINPEEEEEDEDATVS KTRR
OsHVA22l	LOC_Os10g09870	MMGDFLSRVLLLAFGYAYPAYECYKTVELNKPEIEKLIFWCQYWILVALLTVLERFGDFAISW LPLYSEAKLMFFIYLWCPRTKGTSYVYETFFRPYISQYENDIDCSILDLRVRAGDMLVVYWQK VAIIGQTTFFNILKYASAQSPAHSSRSRSTQQSYPQKQQQAQPQQPKQSLPQQQQKQSLPQQ QQQQMPHEKPTTLRRAASAAARTAGIMQQSEDTKIAYSNPKTRRLLPTKSAPTASTRSTVA ATKPVEDLKSSGMKLATEEAPSPSSNAAMPGSEPSAPPLPKSAEDDMSIDEVDIPIEDMDEPV ATPEETPMEEAIRVTRGRLRKRIAAVSTADGGAAN
OsHVA22m	LOC_Os11g05800	MLGELISKVLLVLFGYAMPAFECFKTLETRPDDAHMLRFWCQYWIIVSMVIACESFVSWMP MYGEIKLAFFVYLWYPKTKGSDVVYDSFIRPTVMQYEPNIEQRLEHLRANSGQLIAFYIKNF ADKGTAFFMDFLRYVVSERPEAAANSEPQRSSWSSWNPFASRRQEPSPPPSAPPRERRFSGA DPDDEPPAIADVFRASLGGGAMNRRPHNNNNN
OsHVA22n	LOC_Os11g30500	MGKLWTILTHVHSLAGPTVMLLYPLYASVQAMESPSKLDDEQWLAYWILYSFITLVEMLLES LIYWIPIWYELKLLFIAWLALPNFRGAAFIYNRFVREQLRKHGLAGAGAGAAASVGKKDKS SPSSSPKDKEKTKSKFLSFVTPKKDHEAY
OsHVA22o	LOC_Os11g38920	MGSGSLLKVLAKNFDVLAGPLVALAYPLYASVKAIETKSPVDDQQWLTYWVMYSLITLFELT FASIIQWLPFWPSMKLIFICWLVLPYFNGAAFVYQNYVRPMFVKHQMVNIWYVPQKKGLF GKSDDFLTALDKFIEENGPEALKKLTNKAGKSSKQSGKSWKDSKSSKESKDSKSSKESKEPKP SKDSKQLKPPKVSKESKPLKDSKEDKKAVKEDKKAAAKDSKEQKKALKDSKELKKALKDS KEQGSQKDSDELKPKSNKRVTFAEVEPEKELKASNSDWHPTSEYHSVYPEHNSWSSSFMIFE DENSYWN
OsHVA22p	LOC_Os12g06180	MLGELLSRILLLLFGYAMPAFECFKTVEARPNDAHMLRFWCQYWIIVAMVIAFESLISWMP MYGEIKLAFFVYLWYPKTKGSDVVYDTFLRPIVMQYEPNIEQRLLHLRAKSGQLLSFYMKNF ADKGTAFFMDVLRYVVSDKPEGSNQEDSDAQPAAAACSVVGKKNHPAGNVGGQFLPDD PQQRNKKSGGWSPFATKRRPPSPPRPPQESLFESNPEAAAVAEVLKATINPRPRRGAQNGK NYY
SbHVA22a	SORBI_3001G4357 00	MMGGFLSRVLLLAFGYAYPAYECYKTVELNKPEIEQLIFWCQYWILVALLTVLERFGDFTISW LPFYSEAKLMFFVYLWYPKTKGTTYVYGTFFKPYISQHENEIDRNLLELRARATDMVVIYFQ KAASVGQNTFFDVLKYVAAQSPSQKSRQRPQQESQQPQQQQPQVQVQVQLQQPQPQKQ AAPVMRRAASIAARQAAMAQQSQETKPVPSSPKIKRQTSGKSGSVASTKPAAAASTPKPAG SPKKGEVKPAADPVQTPATSANSPKSEPNAPPLPEAEGVDKMAIDEVSGDAAEGAEELDPA LEETPMEETIRVTRAKLRRRTATEDPAGN

SbHVA22b	SORBI_3002G221800	MGKTWALITHLHALAGPSLTLIYPLYASICAMESTTKVDDEQWLAYWIIYSFITLFEMAAENVLYWIPLWYEAKLLLVAWLVLPQFRGASFIYDKFVREQLRKNGVRLHDHHGHGHGHAADHVPDVFQ
SbHVA22c	SORBI_3002G276500	MAVSFVTRLLTLALGYAYPAYGCYKTLELNAPQMERLRFWCQYWILVAFLTAFESFADCALSWLPMYGEAKLAVVVYLWHPKTMGARHVYDDYLRPFLAAHEADIDRGLVELRARAADATASHLQAAVALGRACLSEVARRVSSQLQQAATSAGPAAQVR
SbHVA22d	SORBI_3003G283500	MGVLGTLARNLDALVGPGIMLLYPLYASMRAIESPSSLDDQQWLTYWVLYSLITLFELSCWKVLQWFPLWPYMKLLFCCWLVLPIFNGAAYIYEAHVRRYFKIGNYVSPIYNERQRRVLQMMSLDARKSVERFIETHGPDALDKIIRAAEEEAKRT
SbHVA22e	SORBI_3003G350600	MALLAPAISGEVGLRLLLAPLSSNVVIRTASCAVGIGLPMYSTFRAIEKKDEKEKERLLLYWAAYGSFSIAEVFADKLLSSVPLYYHVKFAILVWLQFPSNSGSKHVYKRHLRPFFLKHQAKIDRFLNILSKELTKFVSSHEDEIRFIENMAIRGTTTANYIVNGLDQPEETQAANRIEGPNTTVTEETGVSGTET
SbHVA22f	SORBI_3004G018800	MIGSFITGILTLVLGYAYPAYDCYKTVELNRPEVEQLRFWCQYWILLAFLTVLERVGENFVSWLPMYSEAKLAFIVYLWYPKTRGTAYVYESFFKPYIAKHETEIDRNLLELRTRAGDMAVLYFQRVANYAQTRSYEILQYIASQSQTQRSRPQAQQQQQRPPPPRTRQVNPAPPPVPAPSAPPMPPQPAQAQVPPTPMPPPIPVAPPGAVPPAQPPPPPLAPEAAATNGPQDTEAMQVDPPRVSLSGAPPPLPPEETLIEEAIRLTRGRLRRRLVGGSGPPPN
SbHVA22g	SORBI_3004G237200	MGGGSGSFLKVLVNNMDVLAGPLVSLAYPLYASVRAIETKSAVDDQQWLTYWVLYSFITLFELTFAPVLEWLPFWSYAKLFFNCWLVLPQFNGAAHVYEHFVRPMIVNQQVVNIWYIPKKDESGRPDDVISAAQRYIEQNGSKAFENLVNKFKSSNPKRSILEEVEVERRARIQRESEAREVNPFFNPEYQY
SbHVA22h	SORBI_3005G121200	MGKLWTILTHLHSLAGPTVMLLYPLYASVQAMESPSKLDDEQWLAYWILYSFITLMEMVLESLIYWIPIWYELKLLFIAWLALPNFRGAAFIYDKFVREQLRKHGLTTAAGSGSKKDDGKSSSSPSSKDKDKPKSKFLAFVTPKKDHEAY
SbHVA22i	SORBI_3005G121300	MGKLWTILSHLHSLAGPTVTLLYPLYASVQAMESSSKLDDEQWLAYWILYSFITLMEMLLQSLIYWIPVWYELKLLFMAWLVLPNFRGAAFIYNKFVREQVKKHISIQAAGAGSTSNNNVTANKDKKIVSTSPKEKKSKGKLLSMVIPKKLKF
SbHVA22j	SORBI_3005G177900	MGSGSLLKVVANNFDVLAGPLVALAYPLYASVKAIETKSPVDDQQWLTYWVLYSLITLFELTFASIIQWLPFWPSMKLIFICWLVLPYFNGAAYVYQNYVRPAFIKNQMVNIWYVPQKKGLFGKSDDFLTALDKFVEENGTDALKKLANKAGKSFKQSGKSSKDSKESKSSKESKETKSSKDSKE

		LKQSKDAKQPKPSKDLKEPKSPKDPKSPKDSKEQKKAALRDPKKAVKDSKELKKALKDSKQQESLEDPKEHTAKKAGKRVTFAEVEPEKELKASNSDWHPSSDFHSAYPEQNSWASSFMIFEDENSYWNRGHPDC
SbHVA22k	SORBI_3006G073900	MSTELLTKFLTLLFGYAMPALECFKAIEQRPGRADQLRFWCQYWIILVLLVMFDEIAGVLISKIPMYYELKLAFLVYLWYPKTRGTDIVYETFLQPLVMQYQPNIEARLQYLRANAGDILVFYLKNFTERGYDLFLRVLDYVRSQASKGSRTRRFFSFRGERAERPSFADDDYATGGDRRDGGRHRRPRSGY
SbHVA22l	SORBI_3007G153600	MGKSWALVSHLHTIAGPSITLLYPLYASVCAMESPSKVDDEQWLSYWIIYSFITLLEMLAEPLLYWIPVWYPVKLLFVAWLVLPQFKGASFIYEKLVREQLSKYRARYPRKGKGGGGAAAAADVDDDHKVHIAKAEHDHVQ
SbHVA22m	SORBI_3008G042300	MLGELLSKILLLLFGYAMPAFECFKTVETRPNDAHMLRFWCQYWIIVAMVIAVESVISWMPMYSEMKLAFFVYLWYPKTKGSDVVYDTFLRPIVMQYEPNIEQRLLHLRAKSGQLISFYMKNFADKGTAFFMDVLRYVISEKPEGSNAELRNKKSGWSPFATKRRPPSPPPEPLFDSNPDAALLAEALRGAIGAKPRRGSNDKHY
SbHVA22n	SORBI_3010G091900	MGSGSFLKVLANNFDVLAGPLISLAYPLYASVRAIETKNPVDDQQWLTYWVLYSFITLFELTFAPIIEWLPFWSYAKLFFNCWLVLPWFNGAAYVYDHFVRPTFVNRQIVNIWYVPRNEKLGKSDDVLSAAERYIEQNGPEAFEKLISKSTKSSKSRNTKRSILEEAEAENRAKAERESWDENPFYDKNYRY
ZmHVA22a	Zm00001eb009420	MAPPQSTPRKPACPRLPHALPHRADSDGRSPQSLHPLAQLHLAASPTTRASMRAPMEGRPNPRTRSCSFTDTHRRVISFSSTGPGGEEATRRETPEETRKRLEELDALLEGLVEPKMRPPTPPPPPVLTYLRATAIRHRTEDMEQSSVFCSGFSQPMLHKAKGNIFPLLRTSAFPSSHVKEKWAKNLYIASICYGRTVGIIRYASVHAHLGRTGSRRDDLESLAYTLIFLIRGRLPWQGYQLVLPIFNGATYIYEAHVRRYFKIGSYVSPSYSEHHRRVLQMTSFNACRSVERFIDTHGPDALYRIIRAIQGFEDEPPLWRRLASRPDVGLRGRASSLEEAGLAGPLESAGAVLDSQI
ZmHVA22b	Zm00001eb010740	MMGGFLSRVLLLAFGYAYPAYECYKTVELNKPQIEQLIFWCQYWILVALLTVLERFGDFTISWLPFYSEAKVLFFVYLWYPKTKGTTYVYGTFFKPYISQHENEIDRNLLELRARATDMVVLYFHKAASVGQNTFFDVLKYVAAQSPSRKSRLHPHQEPQQQQPQVQVELQQPQPQKQAAPVMRRGASIAARQAAMAQQSLETIPVPSSPKIKRQTSGRPGSVASTKPAAAASTPKPGGSPKKGEVKPAVDPVQTPATSADSPKSEPSAPPLSETEEVDNMAIDEVSGDAAEGAEELDPALEETPMEETIRVTRAKLRRRTATEDSAGN
ZmHVA22c	Zm00001eb037890	MGKSWSLISHLHTVAGPSITLLYPLYASVCAMESPSKADDEQWLSYWIIHSFVTLLEMLAEPLLHWVPVWYPAKVLFAAWLALPQFKGASFVYEKLVREQLRNYRARYPRKGAAAAAVGGDDDDHKVHIAKAEAEHDHVQ
ZmHVA22d	Zm00001eb073940	MFFVYLWYPKTKGTTYVYGTFFKPYISQHENEIDQNLLELRARATDTQPPQQQQPQVQVQQ

		AQPQNQAAPVMRRASSIAARQAAMAQQSQETKPVQSSPKIKRQTSARSGSVASTKPPIAASAPKPGGSPKKGEVKPTADPVQTPTTGADSPKPEPSARSLPGVEGVDKMAIDEASGDAPEGAEELDPALEEETPIEEMIRVTRAKLRRRTTTEVPVGN
ZmHVA22e	Zm00001eb083010	LTAAKVQNSHAEGRAEWRLDLGKASYLNCLLHLSYIEQHESLENIFSSILETTAQAYGEDDNIQPSSGCTDAGQLCHHPVQELDTERASAETAARSGSTLFALFELLALPTFSKLVLPIFNGAAYIYEAHVRHYFKIGNYVSPSYSEHHRRVLQMTSLDVCRSVERFIDTHGPDALDRIIRAEHVVTALSVESYYRVFCIGLLVKHELSLREKNHVSGVSTCVVSPLQCVPSSLWFSLILTTKTVGEALNKPVLGHLAMQKLSGTIQVVLEFFVEIQFDANGKISGAAIRAYLVERSRVCQISDPERNYHCFCMLCSAPSETSMVA
ZmHVA22f	Zm00001eb092150	LGRTGYQLVLPIFNGAAYIYEAHVRRYFKIGSYVSPSYSEHHHRVLQMTSLNARRSVERFIDTHGPDALDRIIRAAEQEAKRT
ZmHVA22g	Zm00001eb100630	MGKTWALITHLHALAGPSLTLIYPLYASICAMESATRVDDEQWLAYWIIYSFITLFEMAAEHVLYWIPLRYEAKLLLAAWLVLPHFRGASFIYDRFVREQLRKNGVSLRGHHHGHAADHVPHVFQAEHRHGVHS
ZmHVA22h	Zm00001eb112930	MGKLWTILTHLHSLAGPTVMLLYPLYASVRAMESPSKLDDEQWLAYWILYSFVTLMEMLLESLIYWIPIWYELKLLFIAWLVLPNFRGAAFIYDKFVREQLRKHGLTAAGSVKSSSPSSKDKDKPKNKFLAFVTPKKDHEAY
ZmHVA22i	Zm00001eb148430	MALLAPAISGEVGLRLLLAPLSSNIVMRTASCAVGIGLPVYSTFRAIEKKDEQEKERMLLYWAAYGSFSIAEVFADKLLSSVPLYYHVKFAILVWLQFPSNSGSKHVYKRYLRPFFLKHQAKIDRFLNILSKELTKFVSSHEDEIRFIENMAIRGATTANHIVNGLDQPEETQAVNTIEGPNTTVTEEAGVSGTET
ZmHVA22j	Zm00001eb208370	MIGSFITGMLTLVLGYAYPAYDCYKTVELNRPEVEQLRFWCQYWILLAFLTVLERVGESFVSWLPMYSEAKLAFIVYLWYPKTRGTAYVYESFFKPYIAKHETEIDRNLLELRTRAGDMAVLYFQRVTNYAQTRSYEILQYIASQSPTQRQAHQQQQRPPPPRTRQVNPAPPPIPAPSAPPMPPQPAQAQVPPTPPRPLVPVVPPGAVPPAPPPTAPEATATNGLQDTETMQVDPPRASLSGPPLPPEETLIEEAIRLTRGRLRRRLAGGSGPPQN
ZmHVA22k	Zm00001eb210700	MFFVYLWYPKTKGTTYVYGTFFKPYISQHENEIDQNLLELRARATDTQPPQQQQPQVQVQQAQPQNQAAPVMRRASSIAARQAAMAQQSQETKPVQSSPKIKRQTSARSGSVASTKPPIAASAPKPGGSPKKGEVKPAADPVQTPTTGADSPKPEPSARSLPGVEGVDKMAIDEASGDAPEGAEELDPALEEETPIEEMIRVTRAKLRRRTTTEVPVGN
ZmHVA22l	Zm00001eb254850	MGGGSGSFLKVLVNNMDVLAGPLVSLAYPLYASVRAIETKSAVDDQQWLTYWVLYSFITLFELTFAPVLEWLPFWSYAKLFFNCWLVLPQFNGAAHVYEHFVRPMIVNQQVVNIWYIPKKDESSRPDDVISAAQRYIEQNGSKAFENLVNKFKSSNPRRSILEEVEVERRARIQLESEAREVNPFFNPEYQY

ZmHVA22m	Zm00001eb372500	MGSGSFLKVLAKNFDVLAGPLISLAYPLYASVRAIETKNPVDDQQWLTYWVLYSFVTLFELTFAPIIEWLPFWSYAKLFFNCWLVLPWFNGAAYVYDHFVRPMFVNRRIVNIWYVPRNEKLGKSDDVLSAAERYIEQNGPEAFEKLISKGITSLFS
ZmHVA22n	Zm00001eb379710	MGSGSFLKVLAKNFDVLAGPVISHAYPLYASVRAIETKNPVDDQQWLTYWVLYSFVTLFELTLAPIIEWLPFWSYAKLFFNYWLVLPWFNVASATTGHIPAAAARAPHHRPLGLTFPAHSAGATAALLPRPDLLQPRFPIALGHRDSLRSDARDVEGNLGARGGRL
ZmHVA22o	Zm00001eb398540	MMGGFLSRVLLLAFGYAYPAYECYKTVELNKPEIAQLIFWCQYWILVALLTVLERFGDFTISWLPFYSEAKLMFFVYLWYPKTKGTTYVYGTFFKPYISQHENEIDRNLLELRARATDTVVLYFQKAASAGQSTFFDVLKYVAAQSPSQKSRQRLHQDSQQPPQQQQPQVQVQQAQPQKQAAPVMRRASSIAARQAAMAQQSQETKPVSSSPKIKRQTSARSGSVASTKPPVAASALKPGGSPKKGEVKPAADPVQTPTTGADSPKPEPSAPSLPGAEGVDKMAIDEASGDAPEGAEELDPALEEETPMEETIRVTRAKLRRRTATEDPAGN
ZmHVA22p	Zm00001eb422010	MSIELLTKFLTLLFGYAMPALECFKAIEQRPGRADQLRFWCEYWIILVLLVMFDEIAGVLISKIPMYYELKLAFLVYLWYPKTRGTDIVYETFLQPLVMQYQPNIEARLQYLRANAGDILVFYLKNFTERGYELFLRVLDYVRSQASRGSRTRSFFSFRGERSERPSFTDDYVTGGDRRDGGRHRRPRSGY
ZmHVA22q	Zm00001eb438800	MMGGFLSRVLLLAFGYAYPAYECYKTVELNKPEIAQLIFWCQYWILVALLTVLERFGDFTISWLPFYSEAKLMFFVYLWYPKTKGTTYVYGTFFKPYISQHENEIDRNLLELRARATDTVVLYFQKAASAGQSTFF
ZmHVA22r	Zm00001eb441760	MIGSFITGMLTLVLGYAYPAYDCYKTVELNRPEVEQLRFWCQYWILLAFLTVLERVGESFVSWLPMYSEAKLAFIVYLWYPKTRGTAYVYESFFKPYIAKHETEIDRNLLELRTRAGDMAVLYFQRVTNYAQTRSYEILQYIASQSPTQRQAHQQQQRPPPPRTRQVNPAPPPIPAPSAPPMPPQPAQAQVPPTPPRPLVPVVPPGAVPPAPPPTAPEATATNGLQDTETMQVDPPRASLSGPPLPPEETLIEEAIRLTRGRLRRRLAGGSGPPQN

Table S13. List of primer sequences used for subcellular localization of selected *SlHVA22* genes

Gene Name	Primer sequence (5' to 3')
SlHVA22a	GCAGCCCGGGGGATCCGAAAAGATGTTGGAGAACTC
	CCATTCTAGAACTAGTGTATATGGACCGGTGAGAGG
SlHVA22f	GCAGCCCGGGGGATCCATGGGTTTGATCAGCTTTCTTCTGA
	CCATTCTAGAACTAGTTCGTCTAATGTACAAGCTTCCT
SlHVA22n	GCAGCCCGGGGGATCCATGGGTCATTTTTGGACTTTGATTT
	CCATTCTAGAACTAGTGTAAGCTTCATGCTCTCCCTTTTTG

Table S14. The primer sequences used in qRT-PCR analysis

Gene Name	Forward Primer	Reverse Primer
SlHVA22a	GAAGAACTCAGATTTTGGTGCC	TGCCAATTTTGCTTCACTGTACA
SlHVA22b	CATCTACATGCTCTAGCCGG	GGTTGAAGAAGCATCTCCATAAG
SlHVA22c	GTACTCACCAAGCAGAAATTCAG	TACTTGCTCTGCTGGCCCTT
SlHVA22d	CTGGAGGAGGTATAGTAAGCT	CATCCACAGGGGATTTGGTC
SlHVA22e	GTGGAGATGCTATTTCAACCC	CTCTGGAGTCATGAAGTGAAC
SlHVA22f	TGCAGACCATCAGTGACATTG	AGTACGAGCAAGAGGGATGC
SlHVA22g	GCTTCGCAGTCAAATCCACC	GATTCAGAAGAAGCACGGGG
SlHVA22h	TCGTGCAGATGATCGACAATG	AACATGCTCCATTGAAGTAAGGT
SlHVA22i	ACTCACCTTCATACACTTGCC	GGAGCATCTCCATAAGAGTGA
SlHVA22j	GCAATTGAGAGCCCTTCACC	GATGAATGGCCAAAATGGTAGC
SlHVA22k	ATCACATGAGGAAGCTCGTCA	CATTAAACCGCGGTATCACCA
SlHVA22l	GGCAAATGTATATGACTCACCTT	TCCTCATCAGAAGTGCTCTCA
SlHVA22m	GCAAATGCTTGAGTATATAGCTTC	GTCCCCTTTGTTGGAACGTC

SlHVA22n	CATCATCATCATCACCACAACAT	AGTAAGCTTCATGCTCTCCCT
SlHVA22o	AAGCCTCACAACCACCTCCA	CTTAGACTCGAAGCTTTGGTG
